# Marine Invertebrates: A Promissory Still Unexplored Source of Inhibitors of Biomedically Relevant Metallo Aminopeptidases Belonging to the M1 and M17 Families

**DOI:** 10.3390/md21050279

**Published:** 2023-04-28

**Authors:** Isel Pascual Alonso, Fabiola Almeida García, Mario Ernesto Valdés Tresanco, Yarini Arrebola Sánchez, Daniel Ojeda del Sol, Belinda Sánchez Ramírez, Isabelle Florent, Marjorie Schmitt, Francesc Xavier Avilés

**Affiliations:** 1 Center for Protein Studies, Faculty of Biology, University of Havana, Havana 10400, Cuba; almeidafabiola0406@gmail.com (F.A.G.); marioe911116@gmail.com (M.E.V.T.); yarini1985@gmail.com (Y.A.S.); dojeda9711@gmail.com (D.O.d.S.); 2 Department of Biological Sciences, University of Calgary, Calgary, AB T2N 1N4, Canada; 3 Centro de Inmunología Molecular, Habana 11600, Cuba; belinda@cim.sld.cu; 4 Unité Molécules de Communication et Adaptation des Microorganismes (MCAM, UMR7245), Muséum National d’Histoire Naturelle, CNRS, CP52, 57 Rue Cuvier, 75005 Paris, France; isabelle.florent@mnhn.fr; 5 Université de Haute-Alsace, Université de Strasbourg, CNRS, LIMA UMR 7042, 68000 Mulhouse, France; marjorie.schmitt@uha.fr; 6 Institute for Biotechnology and Biomedicine and Department of Biochemistry, Universitat Autònoma de Barcelona, 08193 Bellaterra, Spain

**Keywords:** aminopeptidase, aminopeptidase N, aminopeptidase A, TRH-degrading ectoenzyme, leucyl aminopeptidase, enzyme inhibitors, drug-oriented inhibitors, marine invertebrates

## Abstract

Proteolytic enzymes, also known as peptidases, are critical in all living organisms. Peptidases control the cleavage, activation, turnover, and synthesis of proteins and regulate many biochemical and physiological processes. They are also involved in several pathophysiological processes. Among peptidases, aminopeptidases catalyze the cleavage of the N-terminal amino acids of proteins or peptide substrates. They are distributed in many phyla and play critical roles in physiology and pathophysiology. Many of them are metallopeptidases belonging to the M1 and M17 families, among others. Some, such as M1 aminopeptidases N and A, thyrotropin-releasing hormone-degrading ectoenzyme, and M17 leucyl aminopeptidase, are targets for the development of therapeutic agents for human diseases, including cancer, hypertension, central nervous system disorders, inflammation, immune system disorders, skin pathologies, and infectious diseases, such as malaria. The relevance of aminopeptidases has driven the search and identification of potent and selective inhibitors as major tools to control proteolysis with an impact in biochemistry, biotechnology, and biomedicine. The present contribution focuses on marine invertebrate biodiversity as an important and promising source of inhibitors of metalloaminopeptidases from M1 and M17 families, with foreseen biomedical applications in human diseases. The results reviewed in the present contribution support and encourage further studies with inhibitors isolated from marine invertebrates in different biomedical models associated with the activity of these families of exopeptidases.

## 1. Introduction

Proteolytic enzymes, known as peptidases or proteases, are critical in all living organisms [1]. Proteases can act as exo- and/or endopeptidases. They are segregated in classes that strongly depend on the chemical nature of the groups involved in catalysis. The recognized mechanistic classes are aspartic, cysteine, glutamic, metallo, asparagine, mixed, serine, threonine, and a group dedicated to unknown catalytic type [1].

Peptidases are one of the most abundant groups of enzymes in living organisms. Thus, in mammals, more than six hundred genes have been assigned to them. They control the activation, synthesis, and turnover of proteins and regulate most biochemical and physiological processes, such as digestion, fertilization, growth, differentiation, cell signaling/migration, immunological defense, wound healing, and apoptosis [1,2,3]. They are consequently major regulators of homeostasis, ageing, and different human diseases such as cancer, hypertension, diabetes, inflammation, neurodegeneration, and Alzheimer’s disease, among others [4,5,6]. Proteases are also essential for the propagation of infectious agents, being major contributors of pathogenesis in several infectious diseases, including the current coronavirus emergent pandemic SARS COVID-19 [1,7,8,9,10,11,12,13,14].

**Figure 1:**
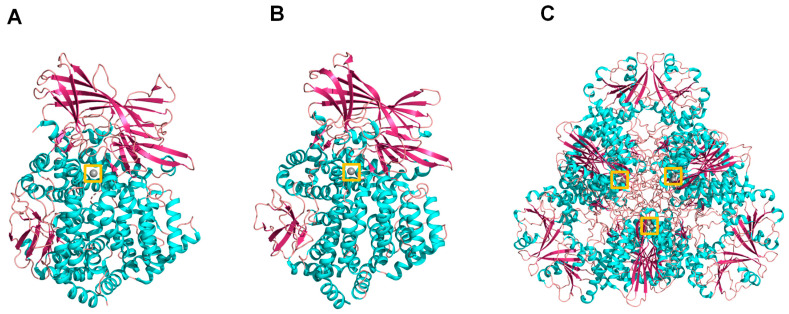
Cartoon representation of the 3D structure of the three aminopeptidases focused on by the present contribution: (**A**) human aminopeptidase N (PDB ID: 4fyq), (**B**) human aminopeptidase A (PDB ID: 4kx7), and (**C**) *Plasmodium* falciparum M17 aminopeptidase PfA-M17 (PDB ID: 4r76). Colors: alpha-helices (cyan), beta sheets (warm pink), and loops (salmon). The zinc atoms are shown as gray spheres highlighted in a yellow box.

Among peptidases, aminopeptidases catalyze the cleavage of the N-terminal amino acids of proteins or peptide substrates. They are distributed in many phyla and play critical roles in physiology and pathophysiology [1,8,9,15]. In mammals, they have a widespread cellular distribution in various organs and are found within cells in many subcellular organelles, in the cytoplasm, as integral membrane proteins, or are exposed or secreted extracellularly [8,16,17,18]. They are mainly metallopeptidases belonging to different families such as M1 and M17, although cysteine and serine peptidases are also included in this group [1]. Among the best representatives of aminopeptidases that are currently in the focus of biomedical investigation, we can pinpoint the M1 family neutral aminopeptidase (APN, EC 3.4.11.2), glutamyl aminopeptidase (APA EC 3.4.11.7) and thyrotropin-releasing hormone-degrading ectoenzyme (TRH-DE; EC 3.4.19.6), and M17 neutral aminopeptidase, also known as leucyl aminopeptidase (LAP, EC 3.4.11.21) (Figure 1). These enzymes are involved in multiple physiological processes as well as in cancer, hypertension, central nervous system disorders, inflammation, immune system disorders, skin pathologies, and infectious diseases, such as malaria, and are current targets for the development of new therapeutic drugs [8,9,19].

**Figure 2:**
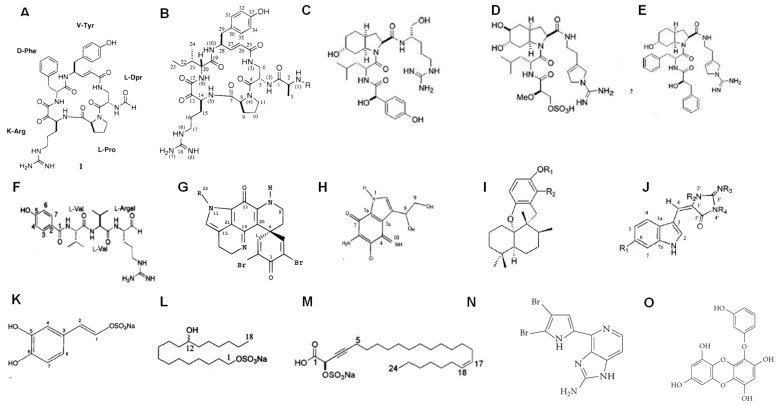
Structures of low molecular weight non-peptidic protease inhibitors of different mechanistic classes isolated from marine organisms. Serine peptidase inhibitors: (**A**) cicloteonamide A [56], (**B**) general structure of cicloteonamide E [56], (**C**) aeruginosine 298-A, (**D**) dinosine, (**E**) oscilarina [57]; cystein peptidase inhibitors: (**F**) tokaramida A [45], (**G**) discorhabdina P [58], (**H**) secobatzellina A [59]; aspartic peptidases inhibitors: (**I**) N,N-dimetiltiocarbamate, (**J**) 6 Br-aplisinopsine [60]; metalloendopeptidase inhibitors: (**K**) jaspisin [43], (**L**) 1-12-hidroxioctadecanil sodium sulphate [61], (**M**) calisponginol sulphate [46], (**N**) Ageladine A [47], (**O**) Eckol [62].

**Figure 3:**
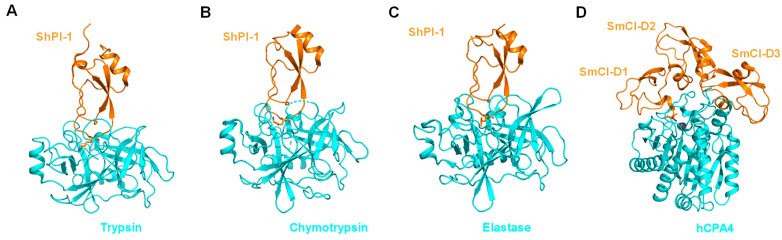
Structures of inhibitors isolated from marine invertebrates in complex with target proteases. (**A**) ShPI-1 isolated from the sea anemone *Stichodactyla helianthus* in complex with bovine trypsin (PDB ID: 3m7q), (**B**) ShPI-1 in complex with bovine chymotrypsin (PDB ID: 3t62), (**C**) ShPI-1 in complex with pancreatic elastase, and (PDB ID: 3m7Q), (**D**) SmCI isolated from marine annelide Sabellastarte magnifica in complex with human carboxypeptidase A4 (hCPA4) [51]. Residues interacting with the S1 subsite have been highlighted as sticks. Proteases and inhibitors are colored cyan and orange, respectively. The zinc atom in hCPA4 is shown as a gray sphere.

**Figure 4:**
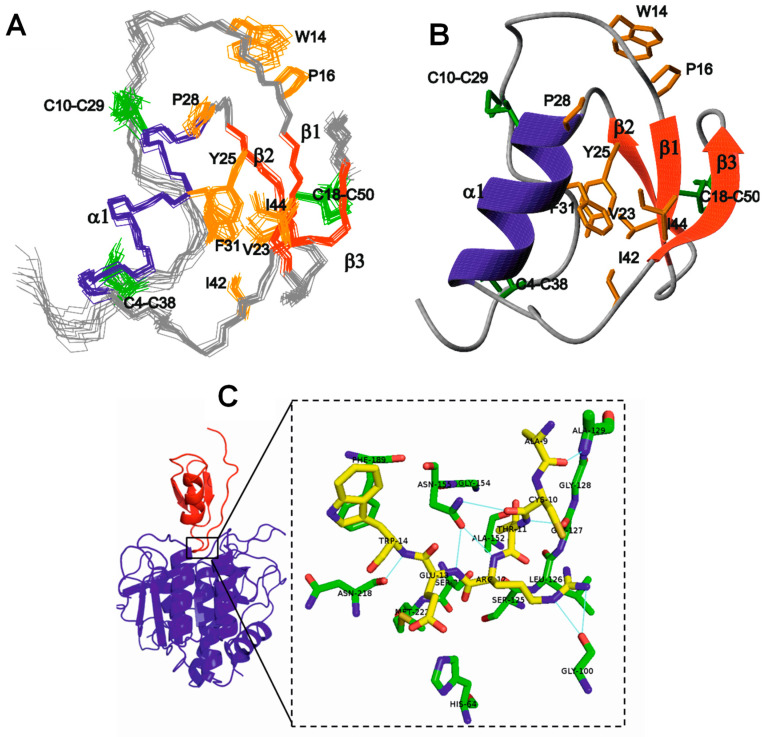
Structure of CmPI-II (a serine protease inhibitor isolated from the marine snail *Cenchritis muricatus*). (**A**) Family of the 15 lowest energy structures of CmPI-II obtained by NMR. (**B**) Ribbons-like representation of the lowest energy structure. The β strands (β1–β3) and the α helix are represented in red and blue, respectively. Orange parts represent the hydrophobic nucleus of the protein and green shows disulfide bridges. (**C**) Prediction of the 3D structure of the CmPI-II/subtilisin A complex. CmPI-II is shown in red and subtilisin A in blue [53]. Courtesy of Prof. Aymara Cabrera Muñoz.

Marine habitats are an extraordinary source of structurally complex bioactive metabolites, characterized by unique functions with marked biological activities and polished through evolution. These features can be attributed to varied environmental conditions, such as access to/lack of light, high pressure, aqueous environment, ionic concentration, pH and temperature changes, scarcity of nutrients, and restricted living spaces. Marine organisms are an abundant source of bioactive molecules (including saccharides, polysaccharides, peptides, proteins, polyketides, polyphenolic compounds, sterol-like products, alkaloids, quinones, and quinolones, among others), such as toxins [20,21], antimicrobial peptides [22,23], antiviral compounds [24], enzymes and enzyme inhibitors [25,26,27,28], and particularly peptidases [29,30] and peptidase inhibitors of almost all mechanistic classes [31,32,33,34,35,36,37,38,39,40,41,42]. These bioactive molecules have a great diversity of chemical structures, high potency, and diverse specificities, especially the inhibitors of metalloenzymes (Figure 2, Figure 3 and Figure 4) [37,43,44,45,46,47,48,49,50,51,52,53,54]. These inhibitory biomolecules are frequently involved in nutrition, homeostasis, reproduction, and communication of marine organisms [27]. Additionally, the high concentration of coexisting organisms in a limited area also makes them very competitive and complex, resulting in the development of adaptations and behaviors aimed at safeguarding the species. Since most invertebrates (e.g., sponges, bryozoans, tunicates, cnidarians, and mollusca snails, among others) lack morphological defense structures, peptidase inhibitors are also part of mechanisms related with protection against predators, infection, and competition [55]. In the present contribution, we review and summarize the status of metalloaminopeptidase inhibitors isolated from marine organisms with a focus on the M1 and M17 families of enzymes targeted in biomedical studies.

## 2. M1 and M17 Metalloexopeptidase Inhibitors Isolated from Marine Invertebrates

### 2.1. Metallopeptidases: General Characteristics and Classification

Metallopeptidases constitute the most diverse catalytic type within proteases, since they include both endopeptidases and exopeptidases, cytosolic enzymes, and others that are secreted to the outside of cells, as well as enzymes associated with the plasma membrane and cell organelles. They are widely distributed in all forms of life such as viruses, bacteria, fungi, and plant and animal cells, indicating the important role they play in biological processes.

Metalloproteases are included among the hydrolases in which the nucleophilic attack on the peptide bond is mediated by a water molecule. This is a feature they share with aspartic-type peptidases, but in metallopeptidases, a divalent metal cation activates the water molecule [63]. This divalent cation is usually zinc (Zn^2+^) but can sometimes be cobalt (Co^2+^) or manganese (Mn^2+^). The metal ion is held in the protein structure by amino acids that act as ligands.

Metallopeptidases can be divided into two large groups based on the number of metal ions required for catalysis. In many metallopeptidases, only one metal ion is required, which frequently is Zn^2+^; however, there is another group of families in which two cocatalytically acting metal ions are required. Within this group, there are families that have two Zn^2+^ ions and all the families in which Co^2+^ or Mn^2+^ are essential for catalysis. In families where only one metal ion acts, three amino acid residues are required to act as metal ligand coordinators, and in families with cocatalytic ions, only five amino acids are required since one of them acts as a ligand coordinator for both metal ions. All metallopeptidases with cocatalytic ions are exopeptidases, while metallopeptidases with a single metal ion can be both exo- and endopeptidases.

Various attempts have been made to classify proteases. The most accepted today is the one initially proposed by Rawlings and Barrett [64,65] that is continuously updated at (https://www.ebi.ac.uk/merops/, accessed on 9 January 2023) [1]. From the general classification of the nine mechanistic classes, they first group the enzymes of each class into families. A family is defined as a group of (homologous) peptidases in which each member shows significant amino acid sequence identity with the “type enzyme” or at least with another member of the family homologous to the type enzyme, mainly in the region of the peptidase that is related to its catalytic activity. The selection criteria used by these authors were very strict, in such a way that they guarantee a common ancestor for the members of a family, which are, therefore, homologous according to the definition of Reeck et al. [66]. Each family is named with a letter denoting the catalytic type (Example: M for metallopeptidases), followed by an arbitrarily assigned number. At a higher level of hierarchy, we find the clan, which is the term used by these authors to describe a group of families, whose members originate from a common ancestor protein but which have diverged to a point where relationships between them cannot be demonstrated by homology in their primary structures. The main evidence for the clan level is the relationship between families in terms of similarities in the three-dimensional structure of their members, in the arrangement of catalytic residues in the peptide structure, as well as similarities in the amino acid sequence around the catalytic residues [1].

Up to now, 16 clans of metallopeptidases have been described: MA, MC, MD, ME, MF, MG, MH, MJ, MM, MN, MO, MP, MQ, MS, MT, and MU, with six of them comprising exo-peptidases. Overall, they form 76 families, with clans MA and MF being two of the most well characterized with enzymes from all living organisms [1].

### 2.2. Clan MA: Subclan MA (E)

**Figure 5:**
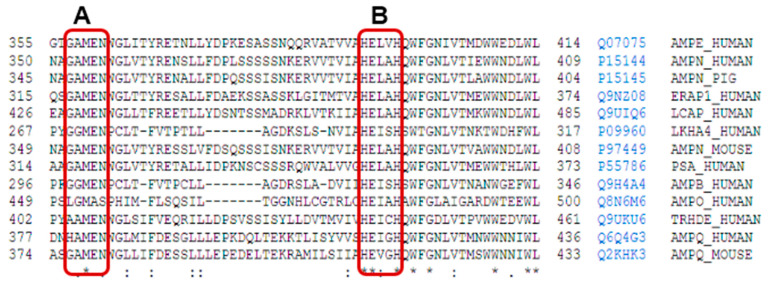
Uniprot alignment of the aminoacidic sequence of the active site of various M1 family aminopeptidases: AMPE_Human: human aminopeptidase E, AMPN_Human: human aminopeptidase N, AMPN_Pig: porcine aminopeptidase N, ERAP1_Human: endoplasmatic reticulum aminopeptidase 1, LCAP_Human: human leucyl-cystinyl aminopeptidase, LKHA4_Human: human leukotriene A4 hydrolase, PSA_Human: human puromycin sensitive aminopeptidase, AMPB_Human: human aminopeptidase B, AMPO_Human: human aminopeptidase O, TRHDE_Human: human thyrotrophin-releasing hormone-degrading enzyme or pyroglutamyl aminopeptidase II, AMPQ_Human: human aminopeptidase Q, AMPQ_Mouse: mouse aminopeptidase Q. On the right of each sequence, the access number and identifiers from Uniprot are included. The short name for each enzyme corresponds to Uniprot abbreviations. The rectangle A encircles the conserved sequence GAMEN related with the aminopeptidase activities of these enzymes from M1 family, and the rectangle B encircles the consensus sequences HEXXH from the active site. Signs below alignment points to other highly conserved amino acid residues inside the M1 family. * indicates residues completely conserved, : indicates position with high degree of conservation of the residues, and . indicates position with mild degree of conservation of the residues.

The clan MA is the largest of the metallopeptidases, with a total of 49 families [1], all consisting of enzymes that contain a single Zn^2+^ in their active sites. This clan is made up of both endopeptidases and exopeptidases, comprising aminopeptidases (families M1, M2, M4, M5, M9, M13, M30, M36, M48, and M61), carboxypeptidases (M2 and M32), peptidyl-dipeptidases (M2), oligopeptidases (M3 and M13), and endopeptidases (families M4, M10, and M12). In the enzymes of the MA clan, the Zn^2+^ atom is coordinated to the protein through two His residues, which are part of the HEXXH motif. In addition to the His residues, the catalytic Zn^2+^ is coordinated by a water molecule and a third residue, the nature of which determines the clan’s subdivision into the MA (E) and MA (M) subclans. In the subclan MA (M), the third ligand can be a residue of His or Asp within the HEXXHXXGXXH/D signature sequence, while in subclan MA (E) the third ligand is a residue of Glu, located at least 14 residues after the carboxyl terminus of the HEXXH motif [1] (Figure 5). The oxygen atom of the water molecule that acts as a metal ligand is the nucleophilic agent that attacks the carbonyl of the peptide bond to be hydrolyzed.

Thermolysin (EC 3.4.24.27), a secretory endopeptidase, is the model enzyme of the MA clan and its structure, widely characterized, is a point of reference for the study of the enzymes of this clan due to the high structural similarity between them in terms of the organization of the active center [1]. Among the most studied families of the subclan MA (E) is M1, whose members show a wide distribution in the living world (Table 1); furthermore, they are involved in many functions that include cell maintenance, growth, development, and defense [8]. This family includes enzymes of Gram (+) and Gram (−) bacteria, cyanobacteria, archaea, protozoa, fungi, animals, and plants [1,8].

### 2.3. M1 Family of Metalloaminopeptidases

The aminopeptidases of the M1 family exist in monomeric or dimeric forms. In eukaryotes, they are generally membrane-associated enzymes such as mammalian APN (i.e., from human or pig), acidic or glutamyl aminopeptidase (APA), adipocyte-derived leucine aminopeptidase, and thyrotropin-releasing hormone-degrading ectoenzyme (TRH-DE), also known as pyroglutamyl peptidase II [16]. Some are cytosolic enzymes, such as leukotriene A4 hydrolase (bifunctional enzyme with aminopeptidase activity) [67] and aminopeptidase B (APB) [68], or associated with the cell wall [9], such as the neutral aminopeptidase (APN, EC 3.4.11.2) of the yeast *Candida albicans* [69]. The structure of the membrane-bound aminopeptidases of the M1 family, in general, comprises a short intracellular tail attached to the transmembrane domain and a large ectodomain formed, in turn, by 2- or 3-folded and conserved domains. Domain I, N-terminal, has a β-sheet nucleus that, although it is widely exposed to the solvent, contains a hydrophobic region that continues in an anchorage region in the membrane. Catalytic domain II, such as that of thermolysin, contains an active site flanked by a mixed structure of β-sheet and α-helix that is highly conserved throughout the family. Domain III, which is composed of an immunoglobulin-like fold, does not appear in some family members (such as leukotriene A4 hydrolase). Domain IV, C-terminal, is the most variable region within the family. It is completely helical, with such an arrangement that it covers the active site; it is also involved in the dimerization of the mammalian isoforms [8]. Disulfide bridges and abundant glycosylations are generally seen in this extracellular region, and some of these enzymes are surface antigens [1,16].

In the M1 family, a well-conserved motif is the Gly-Ala/X-Met-Glu-Asn (GAMEN/GXMEN) sequence. This sequence, also known as the exopeptidase motif, frequently shows variations in the first two residues, and is very useful for the identification of family members [8,16,70] (Figure 5).

**Figure 6:**
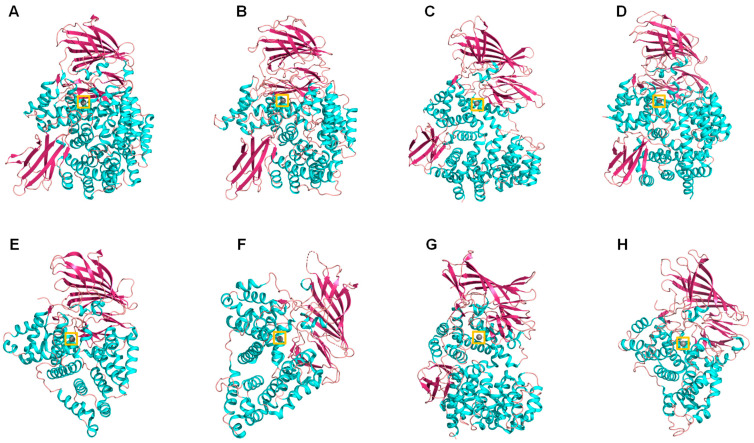
Cartoon representation of different M1 family aminopeptidases: (**A**) pepN from Escherichia coli (PDB ID: 2dq6), (**B**) Plasmodium falciparum aminopeptidase N PfA-M1 (PDB ID: 3ebh), (**C**) human ERAP 1 (PDB ID: 3mdj), (**D**) human ERAP 2 (PDB ID: 3se6), (**E**) human leukotriene A4 hydrolase (PDB ID: 1hs6), (**F**) Saccharomyces cerevisiae leukotriene A4 hydrolase (PDB ID: 2xq0), (**G**) Thermoplasma acidophilum tricorn interacting factor F3 (PDB ID: 1z1w), (**H**) Colwellia psychrerythraea cold-active aminopeptidase (PDB ID: 3cia). Colors: alpha-helices (cyan), beta sheets (warm pink), and loops (salmon). The zinc atoms are shown as gray spheres highlighted in a yellow box.

Through the technique of crystallography and X-ray diffraction, the three-dimensional structures of several members of this family have been elucidated, such as leukotriene A4 hydrolase in complex with its inhibitor bestatin [71]; tricorn-interacting factor 3 of *Thermoplasma acidophilum* [72]; *Escherichia coli* APN (Pep N) in complex with its inhibitor bestatin [73]; *Plasmodium falciparum* (PfA-M1) alone and in complex with bestatin and low molecular mass analogs [74,75,76]; human APA [77]; human ERAP-1 [78]; and porcine and human APN in complex with substrates and bestatin [70,79], among others (Table 2) (Figure 6). In all these structures, it can be seen that the catalytic domain of this enzymatic family presents a high structural similarity with thermolysin, despite the fact that in some cases, there is only 7% identity in sequence with the corresponding polypeptide chains [71]. The high availability of M1 aminopeptidase structures, the well-studied active site able to the binding of small molecules, and the well characterized reaction mechanisms, make M1 aminopeptidases ideal candidates for the application of structure-guided inhibitor discovery, including high-throughput screenings in different databases of marine and other natural compounds. These inhibitors have potentialities in different infectious and chronic human diseases [80,81].

### 2.4. Inhibitors of M1 Family Isolated from Marine Invertebrates

#### 2.4.1. A Specific Inhibitor of Thyrotropin-Releasing Hormone-Degrading Ectoenzyme/Pyroglutamyl Aminopeptidase II Isolated from a Marine Organism

**Figure 7:**
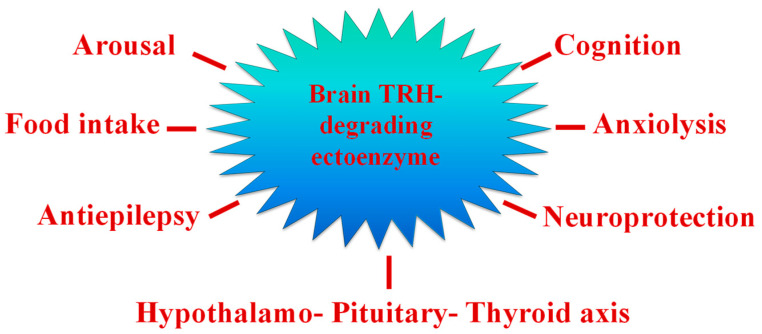
Potential therapeutic applications of targeting central TRH-degrading ectoenzyme activity.

Thyrotropin-releasing hormone (TRH), an N-terminal blocked tripeptide (pGlu-His-ProNH2), is mainly produced by brain neurons. Expressed by neurons of the paraventricular nucleus of the hypothalamus, TRH is a hypophysiotropic factor that increases the synthesis and release of thyroid stimulating hormone (TSH) and prolactin (PRL) from the adenohypophysis. In other central nervous system (CNS) circuits, it functions as a neurotransmitter and/or neuromodulator [82]. This peptide has therapeutic properties in the treatment of brain and spinal damage and various neurodegenerative disorders [83]. However, TRH effects are of short duration, in part because the peptide is hydrolyzed in blood and extracellular space by TRH-DE, the thyrotropin-releasing hormone-degrading ectoenzyme, a M1 family metallopeptidase. TRH-DE is enriched in various brain regions but is also expressed in peripheral tissues including the anterior pituitary and the liver, which secretes a soluble form into blood. Among the M1 metallopeptidases, TRH-DE is the only member with a very narrow specificity, hydrolyzing preferentially the pGlu-His bond of TRH, its best characterized biological substrate, making it a target for the specific manipulation of TRH activity. TRH-DE presents an anatomical location that correlates partially with TRH receptors in various regions and is very strictly regulated by different hormones and hypothalamic factors, as well as by various pharmacological and pathophysiological conditions that alter the transmission of TRH-mediated signals. The regulation of TRH-DE activity may be very important for the adjustment of communication mediated by this peptide [84]. Therefore, TRH-DE inhibitors are important tools for studying the physiological functions of this enzyme and TRH in the CNS, as well as for enhancing the different actions of TRH by protecting the degradation of endogenous TRH or exogenously administered analogues [85]. TRH-DE inhibition may be used to enhance TRH activity in different pathologies (Figure 7). Only a few synthetic PPII inhibitors have been described [86,87,88].

**Figure 8:**
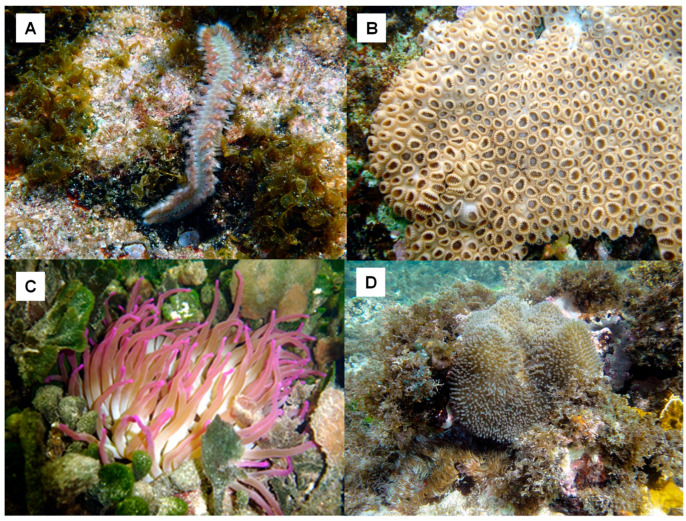
Some of the marine organisms screened for inhibitory activity of TRH-DE and DPP-IV. (**A**) *Hermodice carunculata*, (**B**) *Palythoa caribaeorum*, (**C**) *Condylactis gigantea*, and (**D**) *Stichodactyla helianthus*. Pictures courtesy of Professor José Espinosa, PhD, ICIMAR, CITMA, Cuba.

**Figure 9:**
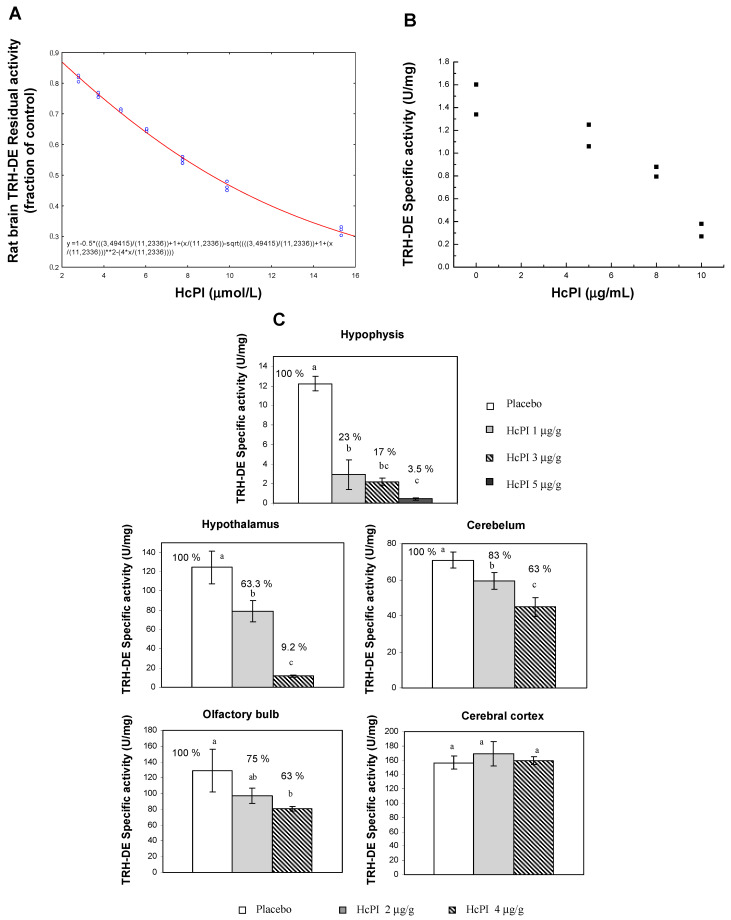
TRH-DE inhibition by HcPI in different enzyme models. (**A**) Ki determination of HcPI effect on TRH-DE activity in rat brain membranes, (**B**) inhibition effect of HcPI on TRH-DE activity in primary cultures of rat adepnohypophysis, and (**C**) effect of intraperitoneal injection of different doses of HcPI on mouse TRH-DE specific activity in brain [49]; Different letters indicate a significant difference between treatment groups, with *p* < 0.001.

A joint project of the Faculty of Biology, University of Havana, Cuba, with the Institute of Biotechnology of UNAM, Mexico, involving a screening in aqueous extracts from 26 Cuban coastline marine organisms (Table 3) resulted in the first natural inhibitor of TRH-DE identified and isolated from the marine annelid *Hermodice carunculata* (Figure 8); it was named HcPI. As a result of this screening, we also detected inhibitory activities of porcine kidney cortex dipeptidyl peptidase IV in the species *Phallusia nigra*, *Mycale microsigmatosa*, *Condylactis gigantea*, *Stichodactyla helianthus*, and *Palythoa caribbaeroum*. HcPI is a 580 Da compound (molecular mass determined by ESI-TOF mass spectrometry), with a possible polymeric structure and the presence of bromine in its structure, as well as amide-type bonds. HcPI potently inhibits TRH-DE with a Ki value of 70.3 nmol/L in a slow and reversible way, making it one of the most powerful inhibitors described against this enzyme [49] (Figure 9A).

Inhibitory specificity studies carried out against proteases of all mechanistic classes indicate that HcPI is highly specific for TRH-DE. The specificity of the inhibitory activity was assayed using several enzymes from each mechanistic class of proteinases. In a concentration range from 33 to 660 ng/mL and preincubation times at 37 °C of 5, 10, or 30 min, HcPI was not active against serine (trypsin, chymotrypsin, elastase, and DPP-IV), cysteine (papain, bromelain, and PPI), or aspartic (pepsin and PR-HIV) proteases nor against metalloproteinases (collagenase, gelatinase, ACE, aminopeptidase N, and carboxypeptidase A). It was further confirmed that HcPI inhibits, in vitro, thyroliberinase, a soluble version of TRH-DE that inactivates TRH in the bloodstream. The inhibition is dose-dependent, with a Ki value of 51 nmol/L, similar to that previously described for TRH-DE. The specificity results support the idea that HcPI should be useful to study the role of TRH-DE in different experimental models. HcPI is not toxic in vivo and its intraperitoneal injection in BalbC mice decreases TRH-DE activity in the pituitary and in different brain regions such as the hypothalamus, cerebellum, and olfactory bulb [49] (Figure 9C). The inhibition of TRH-DE in vivo in this experimental model causes a transient increase in the serum concentrations of prolactin (PRL) and thyrotropin (TSH), which indicates an in vivo enhancement of the actions of endogenous TRH when degradation by TRH-DE is decreased. Additionally, studies on cells were performed. First, in primary cultures of adenohypophyseal cells, 45 min of incubation with HcPI produces a decrease in the activity of membrane-associated TRH-DE, highly dependent on the dose of inhibitor tested with an IC_50_ of 8.3 µg/mL (Figure 9B). Incubation with 8 µg HcPI/mL decreases enzyme activity by 42% from 5 min, an effect stable for at least one hour. Once enzyme inhibition was demonstrated in cultures, the effect of the enzyme on TRH-mediated communication was evaluated, and it was detected that in the absence of TRH in the system, the presence of HcPI (50 µg/mL) does not change the basal levels of secretion of TSH, or PRL. On the other hand, in the presence of TRH (10 nmol/L), the inhibition of TRH-DE by HcPI caused an increase in the levels of PRL released after 30 min by lactotrophs, specialized cells of the adenohypophysis. These results were confirmed in parallel by inhibition of TRH-DE synthesis with the use of antisense RNA, which demonstrated for the first time in a direct way the effects of the regulation of PPII activity on one of the functions of TRH [89]. Intraperitoneal injection of HcPI (1, 5, 20, or 50 µg/g) in mice did not induce any mortality, obvious motor effect, or weight change for up to 15 days. The effect of different doses of HcPI injected intraperitoneally was tested on mouse PPII specific activity. Compared to saline injected animals, PPII activity was significantly decreased 45 min after injection in most of the tissues analyzed; the effect was dose-dependent. Less than 1 mg of the inhibitor per g of animal weight was sufficient to decrease activity by more than 50% in hypophysis; the maximum dose used (5 µg/g) almost completely abolished the activity. The order of potency was as follows: hypophysis > hypothalamus > cerebellum > olfactory bulb (Figure 9) [49]. Other experiments related to the role of PPII in TRH communication within the hypothalamic–adenohypophysis–thyroid axis were continued with the use of animal models. In these studies, HcPI was injected at the beginning of the experiment at a dose of 50 µg/g of animal weight to Wistar rats, dissolved in physiological saline (doses of 5–10 µg/g of animal weight strongly reduce PPII activity in the adenohypophysis and decrease it in CNS regions). The controls received only saline. Four groups of animals were subsequently treated with 1 ng/g animal weight TRH in saline or saline only and slaughtered by decapitation 15 min after the second treatment. Two additional groups were transferred to a cold room kept at 4 °C for 30 min and similarly sacrificed after the end of the experiment. Since cold stress rapidly activates the hypothalamic–adenohypophysis–thyroid axis by increasing concentrations of TRH in the portal hypothalamus–pituitary vessels, this paradigm was used in addition to exogenous administration of TRH, with the objective of evaluating the effects of inhibition of TRH-DE by HcPI on a naturally occurring surge of circulating TSH concentration. Compared to animals that received a single injection of saline, TRH-DE activity is significantly decreased in the hypothalamus and in the pituitary of animals that receive a single dose of HcPI. Similar changes are observed in the activity of serum thyroliberinase. Inhibition of TRH-DE activity by HcPI has no effect on baseline TSH levels, as observed in primary adenohypophyseal cell cultures. However, in animals injected with exogenous TRH or exposed to ambient cold, inhibition of TRH-DE activity by HcPI is associated with a significant increase in serum TSH concentration when compared to control groups that only received saline (Sánchez-Jaramillo et al. 2009). These results demonstrated for the first time the role that TRH-DE exerts on TRH activity and TSH secretion by adenohypophysis and made HcPI a very useful tool for further studies and potential biomedical applications in diseases such as Non-Thyroidal Illness Syndrome (NTIS) in which the levels of thyroid hormones are reduced [90].

#### 2.4.2. Inhibitors of Aminopeptidase N Isolated from Marine Organisms

**Figure 10:**
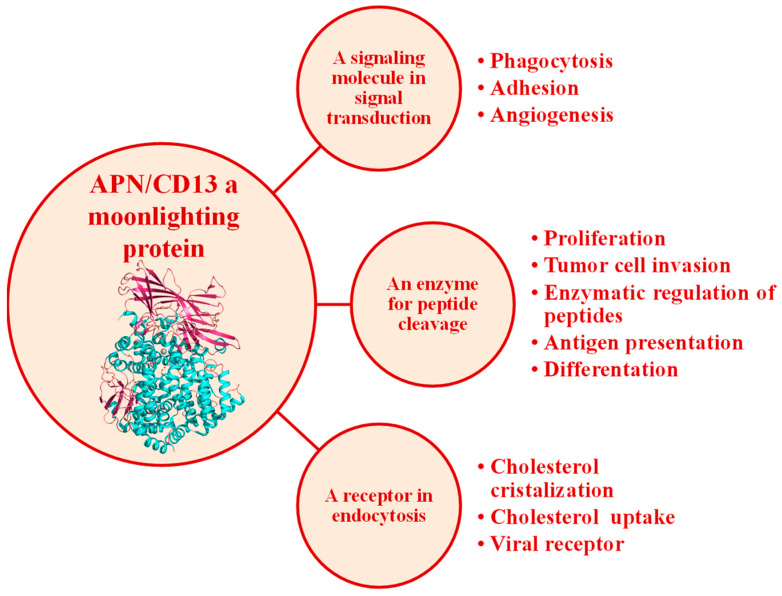
Functions of human APN/CD13 (adapted from Amin et al. [19]).

**Figure 11:**
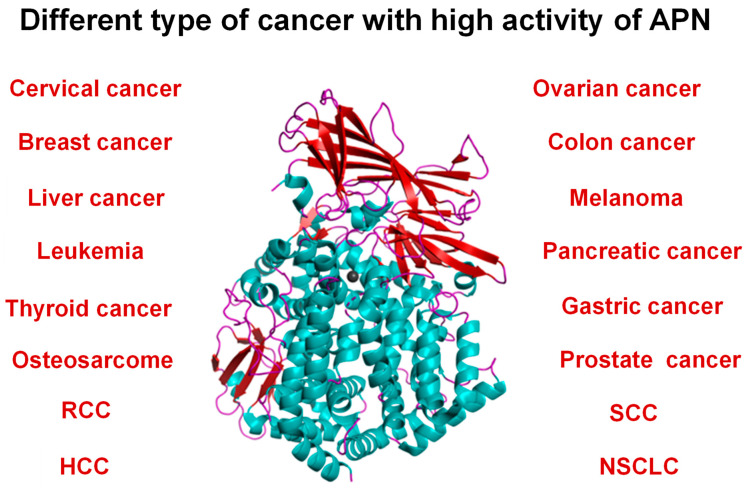
Up-regulation of human APN in different cancers. Abbreviations: renal cell cancer (RCC), hepatocellular carcinoma (HCC), squamous cell carcinoma (SCC), non-small-cell lung carcinoma (NSCLC).

Neutral aminopeptidases are enzymes that catalyze the cleavage of neutral amino acids from the N-terminus of protein or peptide substrates. They have been classified in several metallopeptidase families, such M1 and M17 [1,8,9]. These enzymes are present in all living organisms, but the diversity of the functions in which they are involved is far from being entirely deciphered. Mammalian neutral aminopeptidase (APN, EC 3.4.11.2, M1 family) is the most extensively studied member of the M1 family of zinc-dependent aminopeptidases; it is noteworthy that it catalyzes the cleavage of not only neutral but also basic N-terminal residues. This enzyme, also known as CD13, is widely expressed on cell surfaces of tissues, such as intestinal epithelia and the nervous system. Mammalian APN is a type II membrane protein generally found as a homodimer in several mammalian species. Full-length human APN consists of 967 amino acids with a short N-terminal cytoplasmic domain, a single transmembrane segment, and a large ectodomain containing two catalytic motifs highly conserved across the M1 family: the zinc-binding motif HEXXHX18E and the exopeptidase signature GAMEN [70]. APN plays pivotal roles in many physiological processes, such as pain sensation, sperm motility, cell–cell adhesion, and blood pressure regulation (Figure 10) [8,18]. This enzyme is also up-regulated in human pathologies, such as coronavirus entry, inflammation, immune cell chemotaxis, tumor angiogenesis, and metastasis in several types of cancer, with a strong correlation between the level of APN expression of a cell and its resultant invasive capacity (Figure 10). Dysregulation of APN expression evolves in almost all types of human malignancies, including breast cancer, cervical cancer, ovarian cancer, prostate cancer, non-small-cell lung cancer (NSCLC), liver cancer, colon cancer, cirrhosis gastric cancer, pancreatic cancer, renal cell carcinoma (RCC), hepatocellular carcinoma (HCC), head and neck squamous cell carcinoma (SCC), melanoma, osteosarcoma, and thyroid cancer [19,91]. This makes human APN an attractive target for the treatment of diseases, including cancers (Figure 11) [8,18,19,91,92,93]. Accordingly, strategies for its inhibition have been developed primarily for the treatment of pain [94,95]. Only Ubenimex (bestatin), a drug inhibitor, is currently approved by the FDA for its uses in human pathologies, mainly in cancer [91].

**Figure 12:**
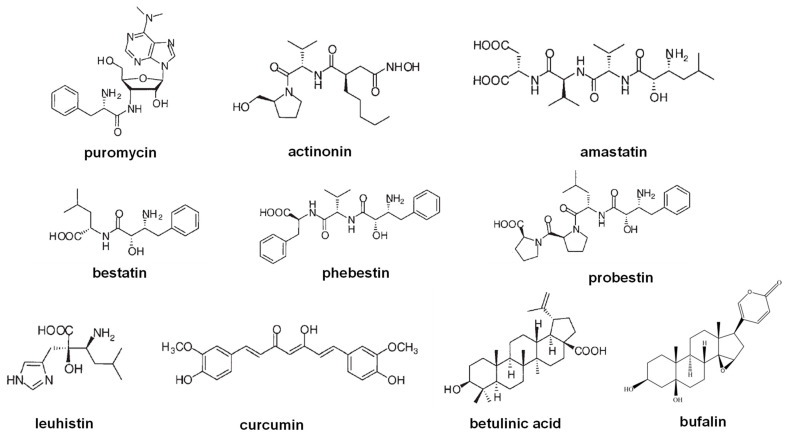
Natural inhibitors of Aminopeptidase N.

Natural inhibitors of human and mammalian APN in general are scarce and have mainly been described from microorganisms [18,96], plants [97], marine invertebrates [36,48,98,99], and more recently from Cuban toad secretions [100,101] (Figure 12). Several compounds from marine organisms have been described with anticancer activities; however, very few of them have been linked with APN inhibition or interaction [102,103,104,105,106]. In the next section, we review the information available regarding marine invertebrates as a promissory and still unexplored source of inhibitors of APN with biomedical relevance mainly in cancer.

##### Psammaplin A

**Figure 13:**
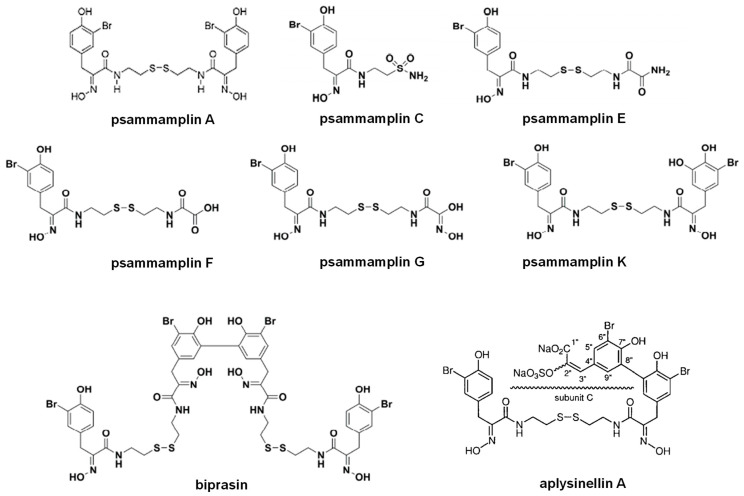
The chemical structure of psammaplin compounds, biprasin, and aplysinellin A.

Psammaplin A (PsA) is a natural bromothyrosine compound belonging to the open-chain-oximinoamidesis bromo-derivates group (Figure 13), isolated from the association between two sponges, *Poecillastra* sp. and *Jaspis* sp. [107], which represents the first isolated natural product containing oxime and disulfide moieties from marine sponges. Subsequently, other natural derivatives, such as biprasin, psammaplin C, psammaplin E, psammaplin F, psammaplin G, and psammaplin K (Figure 13), were also isolated and described [108,109,110,111,112].

Several biological activities have been described for PsA; it is an antibacterial mainly against Staphylococcus aureus (SA) and methicillin-resistant *Staphylococcus aureus* (MRSA) due to DNA gyrase inhibition and bacterial DNA synthesis arrest [113]. PsA also inhibits chitinases that are common in fungi and are crucial for the control of ecdysis in insects [114]. In mammalian cells, PsA inhibits topoisomerase II, an enzyme catalyzing DNA relaxation, with a high IC_50_ of 18.8 mM [114]. In 2004, almost at the same time our group described HcPI as an inhibitor of TRH-DE, Shim et al. [48] found that PsA inhibits mammalian APN with a K_i_ value of 15 µM in a non-competitive way. Structural analogues of PsA, in which phenolic hydroxyl groups were replaced, did not inhibit human or porcine APN, indicating that these groups are crucial in the recognition and inhibition of this enzyme in mammals. This finding perfectly agreed with the effectiveness of bulky and hydrophobic groups in molecules targeting APN [115]. However, no other in-depth structure–activity or docking studies of aminopeptidase N inhibition by PsA have been carried out.

Psammaplin A possesses antiproliferative activities against various cancer cell lines, including triple-negative breast (TNBC, MDA-MB-231), doxorubicin-resistant human breast (MCF-7/adr), colon (HCT15), ovarian (SK-OV-3), lung (A549, LM4175), bone (BoM1833), skin (SK-MEL-2), central nervous system (BrM-2a, XF498) [116,117,118], and Ishikawa endometrial cells [119]. Some of the mechanisms described to explain the antiproliferative effects of this compound are the induction of cell cycle arrest and apoptosis associated with different factors [119,120]. Pretreatment with PsA was also shown to increase the sensitivity of human lung and glioblastoma cancer cells to radiation in vitro [120]. Moreover, this compound showed suppressive effects of the invasion and tube formation of endothelial cells stimulated by the basic fibroblast growth factor [48]. These results demonstrate that PsA is a new APN inhibitor that can be developed as a new antiangiogenic agent.

PsA has also been described as a histone deacetylase (HDAC) inhibitor [119,121]. The mechanism of action underlying the HDAC inhibitory effect of PsA involves a change in the redox state of the disulfide bond. Replacement of the sulfur atom leads to the formation of a mercaptan, which in turn chelates the Zn^+^ ion present in the characteristic active site of the HDAC enzyme, modifying its conformational state and thus preventing its accessibility to the natural substrate. This new conformational state determines an increase in acetylation levels of histone H3, a well-known epigenetic marker of chromatin structure and function, suggesting selectivity for HDACs. Moreover, PsA also exhibits potent enzyme inhibitory and antiproliferative activities under reduced conditions in cells, which indicates that PsA could be used as a natural prodrug [121].

**Figure 14:**
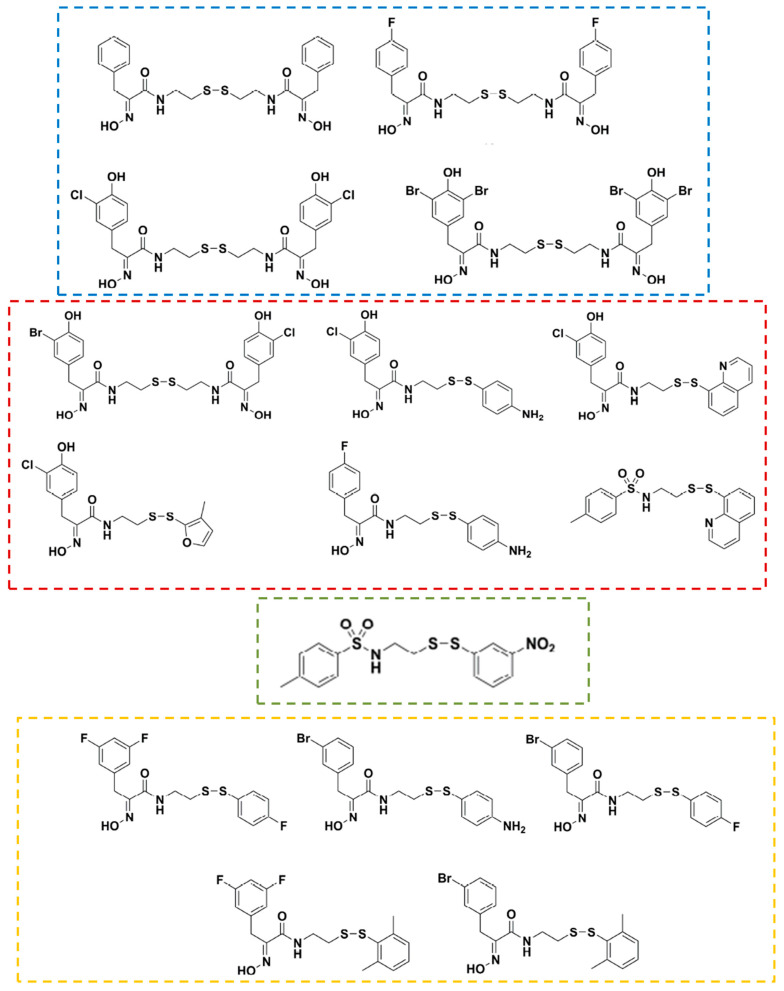
Examples of synthetic homo- and hetero-derivatives of psammaplin A. The blue dashed line box highlights derivatives that showed significant antibacterial effects against methicillin-resistant *Staphylococcus aureus* (MRSA) due to DNA gyrase inhibition and bacterial DNA synthesis arrest. The red dashed line box highlights derivatives that showed higher antibacterial activity than psammaplin A. The green dashed line box highlights a derivative that showed similar antibacterial activity to clinically used drugs vancomycin and ciprofloxacin. The yellow dashed line box highlights derivatives that possessed 50-fold higher activities than psammaplin A vs. *Staphylococcus aureus* and MRSA, in this case mainly by a nonspecific redox-based mechanism (reviewed in [122]).

Although PsA possesses a broad spectrum of bioactivities, its in-depth study has been hindered due to the limited amount of the compound that can be isolated from marine sources as well as its poor physiological stability. For these reasons, homodimeric or heterodimeric analogs of PsA have been obtained by chemical synthesis through a disulfide exchange strategy (Figure 14) [122]. Some of the new synthetic compounds, particularly heterodimeric derivatives, displayed higher antibacterial activity than psammaplin A, comparable to clinically used drugs vancomycin and ciprofloxacin [123,124]. However, the APN inhibitory activity of all the synthetic psammaplin derivatives (examples in Figure 13 and Figure 14) has not been evaluated, being a still unexplored source of new aminopeptidase N inhibitors with biomedical potentialities taking into account the promissory effects of these compounds on several biomedical models [122].

##### Identification of Inhibitory Activity of Mammalian APN in Marine Invertebrates from Cuban Coastline

**Figure 15:**
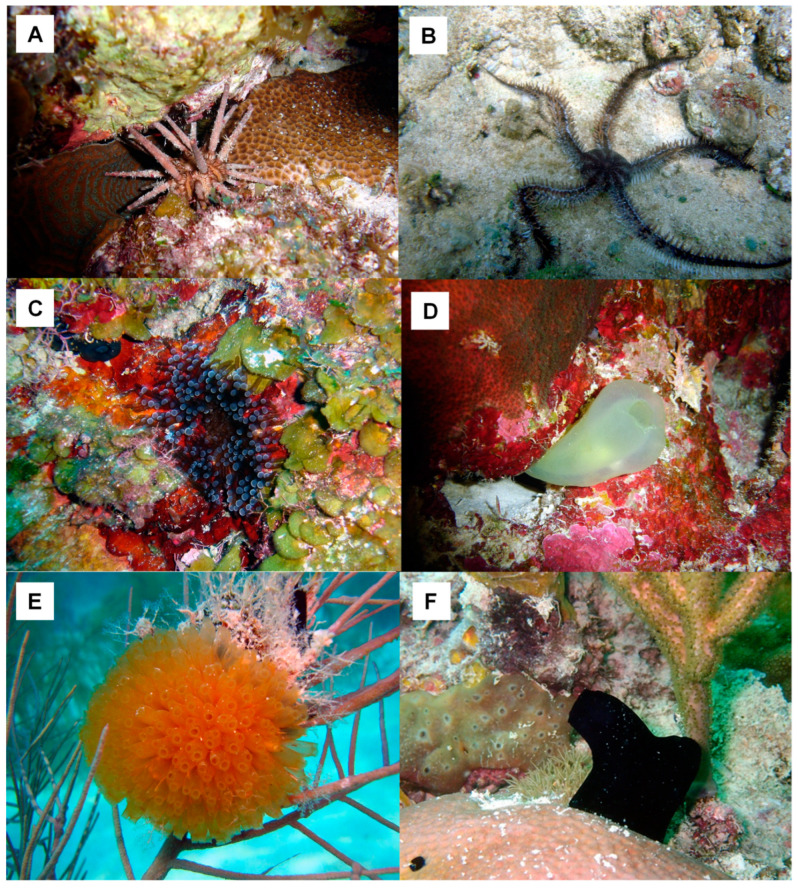
Some of the marine invertebrates screened for inhibitory activity of porcine APN. (**A**) *Eucidiaris tribuloides*, (**B**) *Ophiocoma echinata,* (**C**) *Lebrunia danae*, (**D**) *Ascidia sydneiensis*, (**E**) *Esteinacidia turbinata*, and (**F**) *Phallusia nigra*. Pictures courtesy of Professor José Espinosa, PhD from ICIMAR, CITMA, Cuba.

Considering the identification of a highly specific inhibitor of TRH-DE in the marine annelide *Hermodice carunculata* in aqueous extracts from Cuban marine invertebrates, our group extended the screening to porcine and human APN as targets. As part of a first study carried out in 2011–2015 in aqueous extracts from marine invertebrates belonging to the phyla (Table 4), an APN inhibitory activity is detected in all the extracts evaluated except for *Lebrunia danae* and *Hermodice carunculata*, whose extracts display values of enzymatic activity higher than those of the control test of porcine APN (pAPN) (Table 4, Figure 15). The L-Leu-pNA substrate is also hydrolyzed when the assay is performed only in the presence of the extract and activity buffer, indicating the possible presence of a neutral aminopeptidase type activity in these two species. The extracts of the species *Bryozoo* sp. *2*, *Diplosoma listerianum*, *Lisoclinum verrilli*, *Eucidaris tribuloides*, and *Ophiocoma echinata* were selected as the most promising in terms of the specific inhibitory activity of porcine APN as well as a dose-dependent inhibition behavior [125]. The extracts of the *Phallusia nigra*, *Ascidia sidneyense*, *Microcosmus guanus*, *Steinacidia turbinata*, and *Poticlenum constellatum* species do not show inhibition at increasing concentrations, indicating that the initial result is probably due to a component of the extract that interferes with the correct determination of the enzyme activity. All the selected extracts, except that of *Lisoclinum verrilli*, show slow inhibition, in the order of minutes. On the other hand, for the latter, equilibrium is reached within 1 min of preincubation time, suggesting a fast interaction of the inhibitory components of the extract with the porcine APN. The IC_50_ values are in the range of 0.11–2.39 mg/mL (Table 4), with the highest efficiency being for the extracts of the two sea squirts (*Diplosoma listerianum* and *Lisoclinum verrilli*) and that of *Bryiozoo sp2*. The active extracts were submitted to clarification treatments (such as 2.5% trichloroacetic acid and heat treatments) to eliminate contaminants (mainly proteins) and to promote dissociation from endogenous inhibitor–target complexes. Clarification increases the specific inhibitory activity of the extracts, suggesting that the procedure should be useful in future works dealing with the isolation of the inhibitory molecules [98].

**Figure 16:**
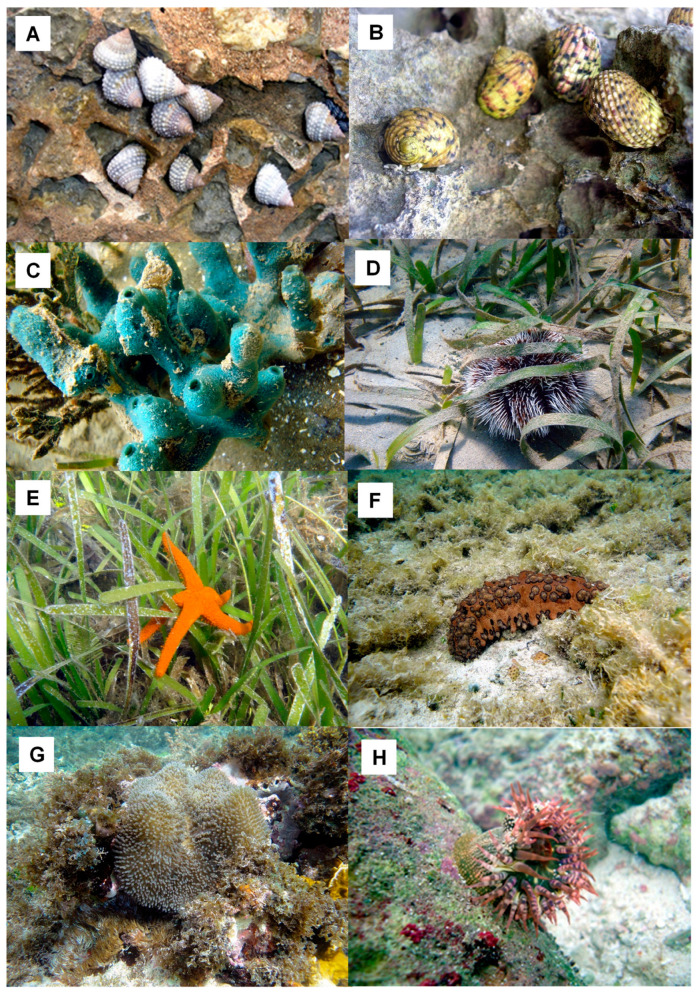
Some of the marine invertebrates which show inhibitory activity of pAPN, pAPA, hAPN, and rPfA-M17. (**A**) *Cenchritis muricatus,* (**B**) *Nerita versicolor*, (**C**) *Lissodendoryx (Lissodendoryx) isodictyalis*, (**D**) *Tripneustes ventricosus*, (**E**) *Echinaster (Othilia) echinophorus*, (**F**) *Isostichopus badionotus*, (**G**) *Stichodactyla helianthus*, and (**H**) *Bunodosoma granuliferum*. Pictures courtesy of Professor José Espinosa, PhD from ICIMAR, CITMA, Cuba.

In a second study performed in 2015–2019, aqueous extracts from species belonging to the phyla Mollusca, Poriphera, Echinodermata, and Cnidaria (Figure 16) were screened using human placental APN as the target (Table 5). The initial evaluations allowed detection of an inhibitory activity of hAPN only from the species *Cenchritis muricatus* and *Isostichopus badionotus*. Increased L-Leu-AMC hydrolysis rates over the control value are found instead of inhibitory activities for the rest of the species. These results suggest either the presence of an activator of the target enzymes used in the assays or neutral aminopeptidase-like enzymes hydrolyzing L-Leu-AMC in the corresponding aqueous extracts. The clarification of all aqueous crude extracts (2.5% TCA treatment) increased, in all cases, the recovery of specific inhibitory activities as compared to their detection in positive crude extracts. The treatment also allowed the identification of inhibitory activities from species that were negative after screening using aqueous crude extracts. This result indicated that clarification eliminates contaminants and/or induces dissociation from endogenous inhibitor–target complexes that do not allow the detection of inhibitory components in crude extracts.

The clarified extracts (2.5% TCA) from the species *Cenchritis muricatus*, *Nerita peloronta*, *Nerita versicolor*, *Lissodendoryx (Lissodendoryx) isodictyalis*, *Tripneustes ventricosus*, *Echinaster (Othilia) echinophorus*, *Isostichopus badionotus*, *Stichodactyla helianthus*, *Bunodosoma granuliferum*, and *Physalia physalis* were used to continue the inhibition studies vs. human APN (hAPN) [98]. Additionally, the enhanced activities over the control detected in some extracts were lost, in all cases, after the 2.5% TCA treatments, suggesting susceptibility of the active molecules to the chaotropic agent and/or to the acidic pH of the molecule(s) responsible for these effects (Table 5).

To test the presence of neutral aminopeptidase-like enzymes in aqueous crude extracts, preliminary enzymatic assays were performed using different amounts of the samples (in absence of the initial target hAPN) in the presence of L-Leu-AMC. A linear dependence of the initial rate versus the amount of crude extract in the assays was detected for the species *Nerita peloronta*, *Nerita versicolor*, *Lissodendoryx (Lissodendoryx) isodictyalis*, *Tripneustes ventricosus*, *Echinaster (Othilia) echinophorus*, *Stichodactyla helianthus*, *Bunodosoma granuliferum*, and *Physalia physalis*. These neutral aminopeptidase-like activities were recently characterized by a kinetic approach combining substrates, inhibitors, and cations, showing for the first time a biochemical behavior indicative of the presence of M1 and M17 enzymes in these species [30].

The clarified extracts inhibit hAPN activity in a dose-dependent manner, and the inhibition was characterized by a concave behavior, indicating the reversibility of the inhibition and corroborating the presence of inhibitory molecules in the samples (and not artifacts interfering with the enzyme activity). The IC_50_ values are in the range of 11.7–567.6 µg/mL [98]. As a promissory result, hAPN is inhibited with IC_50_ values around or less than 100 µg/mL in four of the ten species tested (*Lissodendoryx (Lissodendoryx) isodictyalis*, *Tripneustes ventricosus*, *Isostichopus badionotus*, and *Stichodactyla helianthus*); this inhibition is stronger than that produced by bestatin or amastatin (pure compounds) assayed in parallel as controls (Table 5) [98].

Taking into account that the effect of the inhibition of hAPN was corroborated, the effect of each treated extract on the viability of two APN+ cancer cell lines PC3 and 3LL was evaluated [98]. All treated extracts, and bestatin used as a positive control, have a dose-dependent effect on PC3 and 3LL cell viability. The higher effects on both cell lines, with IC_50_ values below 100 µg/mL, are observed for the species showing the strongest hAPN inhibition. An IC_50_ value under 5 µg/mL for *L. isodictyalis* extract vs. both cancer cells lines, similar to the effect displayed by bestatin, indicates that this species is promissory for the isolation of hAPN inhibitors. In this work, the IC_50_ values for cell viability are in good agreement with the IC_50_ values for hAPN inhibition, including the bestatin results. To the best of our knowledge, this work was the first to show concomitantly natural inhibitor potency on hAPN and indications of activity on hAPN-expressing cells [98].

##### Inhibitors of Aminopeptidase A Isolated from Marine Organisms

**Figure 17:**
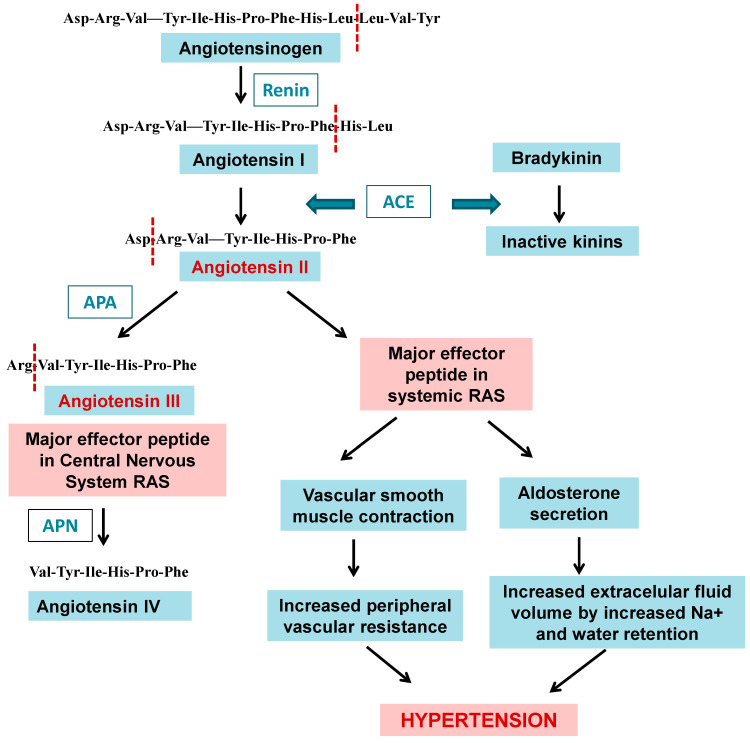
Role of aminopeptidase A and aminopeptidase N in the renin–angiotensin systems.

Membrane glutamyl aminopeptidase, also known as acidic aminopeptidase (APA, EC 3.4.11.7), is a type II membrane protein of the M1 family, MA subclade (E), of metallopeptidases [8] (Figure 1B). This enzyme is widely distributed in mammalian tissues. Aminopeptidase A (APA) has been reported to have molecular weights around 109 kDa for the human and 108 kDa for the porcine enzyme [1]. APA’s S1 pocket accommodates acid residue side chains, whereby this enzyme hydrolyzes aspartic and glutamic residues from the peptide N-terminus [77]. APA performs fundamental functions in a wide range of physiological processes, since it participates in the metabolism of angiotensin II, involved in the renin–angiotensin system in the central nervous system and other anatomical locations, making it an important regulator of blood pressure (Figure 17) [126]. In addition, it is involved in the development of Alzheimer’s disease and glomerulosclerosis and in the progression of cancer. It is associated with the development of renal neoplasms, malignant trophoblasts, renal choriocarcinoma, and colorectal cancer [126,127,128,129,130]. APA plays a key role in blood pressure regulation, which has made it a promising therapeutic target for the development of antihypertensive agents (Figure 17) [30,126,131].

Recently, we extended the screening of marine organism extracts to porcine aminopeptidase A (pAPA). We observed that extracts from the species *Nerita peloronta*, *Nerita versicolor*, *Lissodendoryx (Lissodendoryx) isodictyalis*, *Tripneustes ventricosus*, *Echinaster (Othilia) echinophorus*, *Isostichopus badionotus*, and *Stichodactyla helianthus* displayed dose-dependent inhibition of porcine APA activity, with IC_50_ values in the range of 11.00–1005.00 µg/mL (Table 6), showing that *Nerita versicolor* has a certain selectivity for pAPA rather than for hAPN. These results strongly support the exploration of marine fauna of invertebrates as promissory sources of inhibitors of M1 family enzymes with potential biomedical applications, such as APN and APA.

### 2.5. Clan MF: Family M17

**Figure 18:**
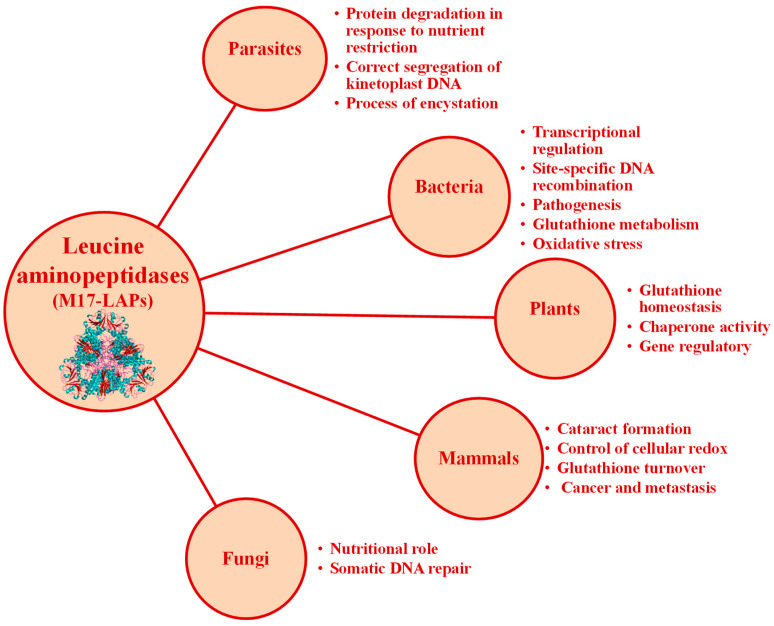
Functions of M17 aminopeptidases from different life groups.

**Figure 19:**
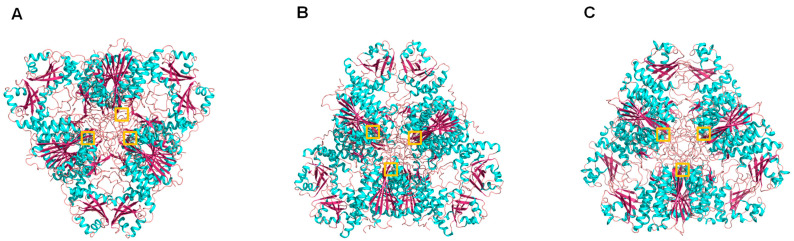
Cartoon representation of different M17 family aminopeptidases: (**A**) *Bos taurus* leucyl aminopeptidase 3 (PDB ID: 1bll), (**B**) *Escherichia coli* PepA aminopeptidase (PDB ID: 1gyt), and (**C**) ZK353.6 (*Caenorhabditis elegans*) (PDB ID: 42hc9). Colors: alpha-helices (cyan), beta sheets (warm pink), and loops (salmon). The zinc atoms are shown as gray spheres highlighted in a yellow box.

Clan MF contains aminopeptidases that require cocatalytic metal ions for activity. The clan contains only the single family M17, a family of leucyl aminopeptidases [1], summarized in Table 7. The M17 aminopeptidases utilize two divalent metal ion cofactors to catalyze the removal of selected N-terminal amino acids from short peptide chains. M17 aminopeptidases are found in all kingdoms (Figure 18), wherein they possess a characteristic homo-hexameric three-dimensional arrangement of their monomers (Figure 19) and play roles in a wide range of cellular processes [9]. The proteolytic reaction contributes to intracellular protein turnover, a fundamental housekeeping process across all living organisms [132] (Figure 18). However, a wide range of additional functions beyond aminopeptidase activity have also been attributed to M17 family members. M17 aminopeptidases from plants possess chaperone activity [133], which might contribute to their function in the stress response pathway [134], while in bacteria they play roles in site-specific DNA recombination [135], and further, can moderate transcription of key virulence factors [136]. Therefore, the family of M17 aminopeptidases is multifunctional, capable of performing diverse organism-specific functions far beyond peptide hydrolysis (Figure 18).

Leucyl aminopeptidases are also distributed in Apicomplexan protist parasites such as *Plasmodium falciparum*, the main agent of malaria in humans. The most important clinical stage of the complex *P. falciparum* life cycle [137], which has attracted the highest attention for the development of antimalarials, takes place in the human erythrocyte, where significant hemoglobin degradation occurs under the concerted action of endo- and exopeptidases [138]. PfA-M17 is involved in the final steps of hemoglobin digestion [139] and is currently a promising chemotherapeutic target as its inhibitors can kill parasites in vitro and in vivo (Figure 1) [9,140,141].

Through the technique of crystallography and X-ray diffraction, the three-dimensional structure of several members of this family has been elucidated, such as leucyl aminopeptidase 3 *from Bos taurus*, leucyl aminopeptidase (plant-type) from *Solanum lycopersicum*, PepA aminopeptidasa from *Escherichia coli* and other bacteria, and PfA-M17 from *Plasmodium falciparum* among others, being PfA-M17, joint with the mammalian enzyme, the more representative in tridimensional structure available at PDB, supporting the relevance of the search for new inhibitors as potential new antimalarial chemotherapy agents (Table 8, Figure 19).

**Figure 20:**
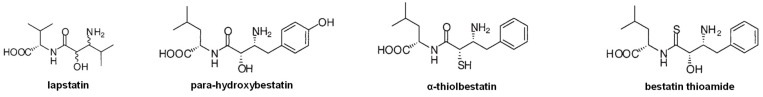
Examples of natural inhibitors of M17 enzymes.

Natural inhibitors of leucine aminopeptidases are scarce and have mainly been described from microorganisms sharing unspecific inhibition of APN and other M1 family inhibitors, such as actinonin, amastatin, bestatin, and various bestatin derivaties (Figure 12 and Figure 20) [18,139,142]. Several compounds from marine organisms have been described with anticancer and antiplasmodial activities [103,106,143,144,145,146]; however, only two of them have been linked with LAP inhibition or interaction [102,104,105]. In the next section, we review the information available regarding marine organisms as a promissory and still unexplored source of inhibitors of M17 enzyme inhibitors with biomedical relevance, mainly in cancer and malaria.

### 2.6. Inhibitors of M17 Leucyl Aminopeptidases from Marine Organisms

In the work of Pascual et al. [98], the screening of inhibitory activities in aqueous extracts from species belonging to the phyla Mollusca, Poriphera, Echinodermata, and Cnidaria from the Cuban coastline involved human LAP (hLAP) and a recombinant form of PfA-M17 (rPfA-M17), both leucil aminopeptidases. As a result of preliminary assays, inhibitory activity vs. hLAP was detected in the species *Cenchritis muricatus*, *Lissodendoryx (Lissodendoryx) isodyctialis*, *Isostichopus badionotus*, and *Stichodactyla helianthus*. Inhibitory activity against rPfA-M17 was detected in the species *Cenchritis muricatus*, *Echinaster (Othilia) echinophorus*, *Isostichopus badionotus*, *Physalia physalis*, *Stichodactyla helianthus*, and *Bunodosoma granuliferum* (specific inhibitory activity values are summarized in Table 9, Figure 16), i.e., in four different phyla. The clarification of all aqueous crude extracts with a 2.5% TCA treatment increased the recovery of specific inhibitory activities as compared to their detection in positive crude extracts. The treatment also allowed the identification of inhibitory activities from species that were negative after screening using aqueous crude extracts. This result indicated that this clarification step was useful in the elimination of contaminants and/or induced dissociation from endogenous inhibitor–target complexes that did not allow the detection of inhibitory components in crude extracts.

All the clarified extracts showed inhibitory activity against both peptidases: malarial rPfA-M17 and native human LAP (Table 10, Figure 16). These activities have a concave dose-response behavior, corroborating the presence of reversible inhibitory molecules with IC_50_ values in µg/mL for both enzymes tested (Table 10). Inhibition of rPfA-M17 with IC_50_ values up to ~100 µg/mL is detected for 6 of the 10 extracts (those of *Cenchritis muricatus*, *Nerita perolonta*, *Lissodendoryx (Lissodendoryx) isodictyalis*, *Tripneustes ventricosus*, *Isostichopus badionotus*, and *Stichodactyla helianthus*). Comparing the inhibitions on rPfA-M17 and hLAP, in all cases, the plasmodial enzyme was more susceptible than its human counterpart, with ratios of selectivity between 1.87 and 60 times. The most selective extract was from *Nerita versicolor*, an attractive result even if its IC_50_ value for the malarial enzyme is moderate. All of the treated extracts displayed a dose-dependent effect on a chloroquine-resistant *Plasmodium falciparum* strain (FcB1) cell’s viability, with the exception of *Cenchritis muricatus*, *Stichodactyla helianthus*, and *Bunodosoma granuliferum*. The best effects were obtained for *Tripneustes ventricosus* and *Lissodendoryx (Lissodendoryx) isodictyalis*. Particularly attractive were the *T. ventricosus* extracts that displayed a 300 times more potent effect on the FcB1 strain of *P. falciparum* than the human cancer cells, indicating parasite effect specificity. Additionally, this effect of the *T. ventricosus* extract on the FcB1 strain was stronger than the effect of bestatin (a pure compound). Due to the fact that IC_50_ for *L. isodyctialis* and *T. ventricosus* were lower on the FcB1 strain of *P. falciparum* than on the PfA-M17 recombinant enzyme, it is very likely that these extracts may contain not only PfA-M17 inhibitors but also other compounds active on other malarial targets. For example, for *L. isodyctialis*, inhibition of subtilisin from *Bacillus licheniformes* with an IC_50_ value of 3 µg/mL was described by Gonzalez et al. [39]. Another interesting result is the selectivity for rPfA-M17 regarding hLAP of *Nerita versicolor* extract showing effects on parasite growth with IC_50_ in the same order of enzyme inhibition, indicating that this species is also attractive. These results are the first and still only report of inhibition of M17 enzymes (human and plasmodial) by marine invertebrate species aqueous extracts and supports that sponges as well as marine invertebrates from other phyla such as mollusks, echinoderms, and cnidarians are a good and still underexplored source of potential anticancer and antimalarials associated with the inhibition of neutral aminopeptidases from the M1 and M17 families involved in these human pathologies.

**Figure 21:**
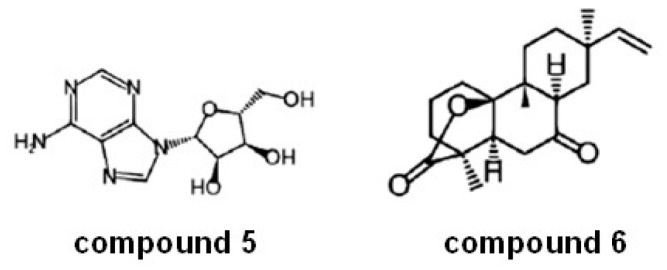
Compounds **5** and **6** as new inhibitors of LAP3 (adapted from Yang et al. [149]).

M17 enzymes are not only a very well established target for malaria and other parasitic diseases but also for bacterial infections and chronic pathologies such as cancer. Bacterial LAPs (from gram-negative or -positive bacteria such as *Escherichia coli*, *Aeromonas proteolytica, Streptomyces lividans*, and *Pseudomonas aeruginosa*, among others) are important virulence factors [147]. Leucine aminopeptidase 3 in humans (LAP3) is associated with various diseases and cancers, such as breast cancer and ovarian cancer [148]. Recently, Yang et al. [149] identified two compounds named compounds 5 and 6 (Figure 21) from 43 natural marine products screened as new inhibitors of LAP3 (from K562 cells with overexpression of LAP3). The inhibition of LAP3 at 30 µM by these two compounds was stronger than that of bestatin used as a control of inhibition in the same conditions. The authors explored the anticancer properties of these new compounds in different models of breast cancer. The results showed that compounds 5 and 6 displayed stronger antiproliferative activity of the breast cancer tumor cells MDA-MB-231 at 30 µM than bestatin. Additionally, both compounds 5 and 6 displayed a more potent suppression effect on the migration of MDA-MB-231 cells than bestatin (the effect of compound **5** was stronger than that of compound **6**). It is well established that LAP3 plays an important role in the metastasis of breast cancer; hence LAP3 inhibitors may have a remarkable effect on the treatment of breast cancer [149].

## 3. Conclusions

Marine biodiversity is an important and promising source of inhibitors of metalloexopeptidases from different families, in particular M1 and M17 enzymes with biomedical applications in human diseases. The results reviewed in the present contribution support and encourage further fundamental applicative studies with inhibitors isolated from marine species in different biomedical models associated with the activity of these families of exopeptidases.

## Figures and Tables

**Table 1:** Some members of the M1 family of the MA(E) subclan of metallopeptidases. (Compiled from https://www.ebi.ac.uk/merops/cgi-bin/, accessed on 9 January 2023).

Enzyme	IUBMB * Nomenclature	Merops ID	Sources
Aminopeptidase N (APN)	EC 3.4.11.2	M01.001	*Homo sapiens, Sus scrofa*
Lysyl aminopeptidase	-	M01.002	*Escherichia coli*
Aminopeptidase A (APA)	EC 3.4.11.7	M01.003	*Homo sapiens*
Leukotriene A4 hydrolase (LTA4H)	EC 3.3.2.6	M01.004	*Homo sapiens*
Alanyl aminopeptidase (bacterial-type)	EC 3.4.11.2	M01.005	*Escherichia coli, Arabidopsis thaliana*
Ape2 aminopeptidase	-	M01.006	*Saccharomyces cerevisiae*
Aap1’ aminopeptidase	-	M01.007	*Saccharomyces cerevisiae*
Thyrotropin-releasing hormone-degrading ectoenzyme or Pyroglutamyl-peptidase II (TRH-DE, PPII)	EC 3.4.19.6	M01.008	*Homo sapiens*, *Mus musculus*, *Ratus novergicus*
Aminopeptidase N (actinomycete-type)	-	M01.009	*Streptomyces lividans*
Cytosol alanyl aminopeptidase	-	M01.010	*Homo sapiens*, *Arabidopsis thaliana*, *Caenorhabditis elegans*
Insulin-regulated membrane aminopeptidase or cystinyl Aminopeptidase (IRAP)	EC 3.4.11.3	M01.011	*Homo sapiens*
Aminopeptidase G	-	M01.012	*Streptomyces coelicolor*
Aminopeptidase N (insect)	-	M01.013	*Manduca sexta*
Aminopeptidase B (APB)	EC 3.4.11.6	M01.014	*Homo sapiens*
Aminopeptidase H11 (nematode)	-	M01.015	
Aminopeptidase Ey	EC 3.4.11.20	M01.016	*Gallus gallus domesticus*
TMA108 protein	-	M01.017	*Saccharomyces cerevisiae*
Endoplasmic reticulum aminopeptidase 1 ERAP-1	-	M01.018	*Homo sapiens*
Tricorn interacting factor F2	-	M01.020	*Thermoplasma acidophilum*
Tricorn interacting factor F3	-	M01.021	*Thermoplasma acidophilum*
Arginyl aminopeptidase-like 1	-	M01.022	*Homo sapiens*
Endoplasmic reticulum aminopeptidase 2 ERAP-2	-	M01.024	*Homo sapiens*
Aminopeptidase-1 (Caenorhabditis-type)	-	M01.025	*Caenorhabditis elegans*
Aminopeptidase Q	-	M01.026	*Homo sapiens*
Aminopeptidase O (AP-O)	EC 3.4.11.-	M01.028	*Homo sapiens*
M1 aminopeptidase (*Plasmodium* spp.)	EC 3.4.11.2	M01.029	*Plasmodium falciparum*
Aminopeptidase N2 (insect)	-	M01.030	*Manduca sexta*
Cold-active aminopeptidase (*Colwellia psychrerythraea*)-Type peptidase	-	M01.031	*Colwellia psychrerythraea*
Lysyl aminopeptidase 1 (*Streptomyces* sp.)	-	M01.032	*Streptomyces albulus*
Lysyl endopeptidase (*Streptomyces albulus*)	-	M01.033	*Streptomyces albulus*
Leukotriene A4 hydrolase (*Saccharomyces cerevisiae*)	EC 3.3.2.6	M01.034	*Saccharomyces cerevisiae*
LePepA g.p. (*Legionella pneumophila*)	-	M01.035	*Legionella pneumophila*

* IUBMB: International Union of Biochemistry and Molecular Biology.

**Table 2:** Crystallographic structures reported for members of the M1 family of metallopeptidases (Compiled from https://www.ebi.ac.uk/merops/cgi-bin/, accessed on 10 January 2023).

Enzyme	Source	Crystallographic Codes of the Structures at Protein Data Bank (PDB)
aminopeptidase N	*Plasmodium falciparum*	3EBG, 3EBH, 3EBI, 3Q43, 3Q44, 3T8V, 4J3B, 4K5L, 4K5M, 4K5N, 4K5O,4K5P, 4R5T, 4R5V, 4R5X, 4ZQT, 4ZW3, 4ZW5, 4ZW6, 4ZW7, 4ZW8, 4ZX3, 4ZX4, 4ZX5, 4ZX6, 5XM7, 5Y19, 5Y1H, 5Y1K, 5Y1Q, 5Y1R, 5Y1S, 5Y1T, 5Y1V, 5Y1W, 5Y1X, 6EA1, 6EA2, 6EAA, 6EAB, 6EE3, 6EE4, 6EE6, 6EED, 6SBQ, 6SBR
aminopeptidase N	*Escherichia coli*	2DQ6, 2DQM, 2HPO, 2HPT, 2ZXG, 3B2P, 3B2X, 3B34, 3B37, 3B3B, 3KED, 3PUU, 3QJX,4Q4E, 4Q4I, 4XMT, 4XMU, 4XMV, 4XMW, 4XMX, 4XM2, 4XN1, 4XN2, 4XN4, 4XN5, 4XN7, 4XN8, 4XN9, 4XNA, 4XNB, 4XND, 4X03, 4X04, 4X05, 5MFR, 5MFS, 5MFT, 5Y01, 5YQ1, 5YQ2, 5YQB, 6G8B
aminopeptidase N	*Homo sapiens*	4FYQ, 4FYR, 4FYS, 4FYT, 5LHD, 6AKT
aminopeptidase N	*Sus scrofa*	4FSC, 4FKE, 4FKH, 4FKK, 4HOM, 4NAQ, 4NZ8, 4OU3
ERAP 1	*Homo sapiens*	2XDT, 2YD0, 3MDJ, 3QNF, 3RJO, 6Q4R
ERAP 2	*Homo sapiens*	3SE6, 4E36, 4JBS, 5AB0, 5AB2, 5CU5, 5J6S, 5KIV
aminopeptidase A	*Homo sapiens*	4KX7, 4KX8, 4KX9, 4KXA, 4KXB, 4KXC, 4KXD
leukotriene A4 hydrolase	*Homo sapiens*	1G6W, 1H19, 1HS6, 1SQM, 2R59, 2VJ8, 3B7R, 3B7S, 3B7T, 3B7U, 3CHO, 3CHP, 3CHQ, 3CHR, 3CHS, 3FH5, 3FH7, 3FH8, 3FHE, 3FTS, 3FTU, 3FTV, 3FTW, 3FTX, 3FTY, 3FTZ, 3FU0, 3FU3, 3FU5, 3FU6, 3FUD, 3FUE, 3FUF, 3FUH, 3FUI, 3FUJ, 3FUK, 3FUL, 3FUM, 3FUN, 3U9W, 4DPR, 4L2L, 4MKT, 4Ms6, 4RSY, 4RVB, 5AEN, 5BPP, 5FWQ, 5N3W, 5NI2, 5NI4, 5NI6, 5NIA, 5NID, 5NIE, 6ENB, 6ENC, 6END
leukotriene A4 hydrolase (S*accharomyces cerevisiae*)	*Saccharomyces cerevisiae* (ATCC 204508/S288c)	2XPY, 2XPZ, 2XQ0
cold-active aminopeptidase (*Colwellia psychrerythraea*)-type peptidase	*Colwellia psychrerythraea* (34H/ATCC BAA-681)	3CIA
tricorn interacting factor F3	*Thermoplasma acidophilum* (ATCC 25905/DSM 1728/JCM 9062/NBRC 15155/AMRC-C165)	1Z1W,1Z5H, 3Q7J
LePepA g.p. (*Legionella pneumophila*)	*Legionella pneumophila*	5ZI5, 5ZI7, 5ZIE
TATA-binding protein-associated factor	*Homo sapiens*	5FUR, 6MZC, 6MZL, 6MZM
IRAP (cystinil aminopeptidase)	*Homo sapiens*	4P8Q, 4PJ6, 4Z7I, 5C97, 5MJ6

**Table 3:** Screening of inhibitory activity of TRH-DE and DPP-IV in aqueous extracts from marine invertebrates collected at the Havana coastline, Cuba (adapted from Pascual et al. [49]).

Species	Phylum	[Protein] Crude Extract (mg/mL)	Inhibit Activity of DPP-IV (U/mg)	Inhibit Activity of TRH-DE (U/mg)
*Caulerpa racemosa*	*Chlorophyta*	5.86	-	-
*Dictyosphaeria cavernosa*	*Chlorophycota*	8.73	-	-
*Halimeda opuntia*	*Chlorophycota*	14.72	-	-
*Halimeda incrassata*	*Chlorophycota*	10.23	-	-
*Bidens pilosa*	*Magnoliophyta*	22.78	-	-
*Ascidia sidneyense*	*Chordata*	57.27	-	-
*Molgula occidentalis*	*Chordata*	31.41	-	-
*Pyura vittata*	*Chordata*	58.53	-	-
*Phallusia nigra*	*Chordata*	25.90	93.00	-
*Microcosmus gamus*	*Chordata*	24.45	-	-
*Tectitethya cripta*	*Porifera*	0.90	-	-
*Mycale microsigmatosa*	*Porifera*	43.50	56.59	-
*Lima scabra*	*Mollusca*	51.55	-	-
*Aplisia dactilomela*	*Mollusca*	24.00	-	-
*Zoanthus pullchelus*	*Cnidaria*	15.57	-	-
*Plexaura homomalla*	Cnidaria	15.25	-	-
*Condylactis gigantea*	*Cnidaria*	36.60	79.46	-
*Stichodactyla helianthus*	*Cnidaria*	79.50	17.48	-
*Cassiopea xamachana*	*Cnidaria*	3.14	-	-
*Physalia physalis*	*Cnidaria*	13.84	-	-
*Palythoa caribaeorum*	*Cnidaria*	16.80	133.00	-
*Bartholomea annulata*	*Cnidaria*	56.50		-
*Hermodice carunculata*	*Annelida*	62.41	-	24.00
*Sabellastarte magnifica*	*Annelida*	67.24	-	-
*Holothuria floridiana*	*Echinodermata*	18.84	-	-
*Holothuria mexicana*	*Echinodermata*	29.63	-	-

**Table 4:** Summary of the preliminary characterization of the porcine APN inhibitory activity detected in crude extracts of marine invertebrate species (screening in the period 2011–2015) (adapted from Pascual et al. [125]).

Species	Phylum	pAPN Inhibitory Activity (U/mL)	pAPN Specific Inhibitory Activity (U/mg)	Pre-Incubation Time (min)	IC_50_ (mg/mL)
*Phallusia nigra*	Chordata	2.3060	0.8363	-	-
*Lisoclinum verrilli*	Chordata	7.8770	0.7361	1	0.11 ± 0.06
*Ascidia sidneyensis*	Chordata	3.1760	0.6481	-	-
*Microcosmus guanus*	Chordata	7.1368	0.5366	-	-
*Esteinacidia turbinata*	Chordata	3.6340	0.5344	-	-
*Diplosoma listerianum*	Chordata	8.1300	0.4394	30	0.11 ± 0.26
*Poticlenum constellatum*	Chordata	3.2230	0.2984	-	-
*Eucidaris tribuloides*	Echinodermata	6.2526	0.3206	5	1.35 ± 0.19
*Ophiocoma echinata*	Echinodermata	8.2337	0.0874	60	2.39 ± 1.09
*Lebrunia danae*	Echinodermata	-	-	-	-
*Bryozoo* sp1	Bryozoa	5.7240	0.0560	-	-
*Bryozoo* sp2	Bryozoa	6.7600	1.1266	30	0.29 ± 0.05
*Hermodice carunculata*	Annelida	-	-	-	-

**Table 5:** Summary of the screening of phyla Mollusca, Poriphera, Echinodermata, and Cnidaria for inhibitory activity against human APN (hAPN) (see note below). Adapted from [98].

Species	Phylum	NA-like Activity (×10^4^ U/mg)	Crude Extracts hAPN sIA (U/mg)	2.5% TCA Treated Extracts hAPN sIA (U/mg)
*Cenchritis muricatus*	Mollusca	ND	0.46	5.20
*Nerita peloronta*	Mollusca	6.95 ± 2.84	ND	5.06
*Nerita versicolor*	Mollusca	12.56 ± 1.70	ND	2.21
*Lissodendoryx (Lissodendoryx) isodictyalis*	Porifera	1.51 ± 0.49	ND	171.92
*Tripneustes ventricosus*	Echinodermata	6.39 ± 0.07	ND	56.86
*Echinaster (Othilia) echinophorus*	Echinodermata	58.87 ± 12.62	ND	10.82
*Isostichopus badionotus*	Echinodermata	ND	1.56	33.81
*Stichodactyla helianthus*	Cnidaria	9.63 ± 2.68	ND	32.81
*Bunodosoma granuliferum*	Cnidaria	39.44 ± 5.45	ND	4.31
*Physalia physalis*	Cnidaria	2.04 ± 0.52	ND	13.13

NOTE: hAPN inhibitory activities in aqueous crude and 2.5% TCA extracts are expressed as specific Inhibitory Activity (sIA) in U/mg. One unit of enzyme activity was defined as the amount of enzyme needed to produce one arbitrary unit of fluorescence per minute, and inhibitory activities are expressed per mg of extract. The first column indicates tested species, the second column shows neutral aminopeptidase-like activity (NA) detected in aqueous crude extracts using L-Leu-AMC as substrate, and the remaining columns refer to sIA. (ND): not detected.

**Table 6:** Summary of the preliminary characterization of inhibitory activities against porcine APA, porcine APN, and human APN in TCA 2.5% treated crude extracts from marine invertebrates (screening in the period 2015–2019). Effect on 3LL and PC3 tumor cell viability (adapted from [98]). ND: not determined.

Species	Phylum	IC_50_ vs. pAPA (µg/mL)	IC_50_ vs. pAPN (µg/ML)	IC_50_ vs. hAPN (µg/mL)	IC_50_ vs. 3LL Viability (µg/mL)	IC_50_ vs. PC3 Viability (µg/mL)
*Cenchritis muricatus*	Mollusca	ND	ND	450.20 ± 77.40	214.00 ± 46.50	352.90 ± 65.00
*Nerita peloronta*	Mollusca	487 ± 11.03	287.03 ± 12.00	237.80 ± 20.90	273.30 ± 78.80	299.10 ± 31.70
*Nerita versicolor*	Mollusca	92.23 ± 12.34	132.11 ± 22.05	370.00 ± 50.00	358.80 ± 70.20	289.70 ± 39.70
*Lissodendoryx (Lissodendoryx) isodictyalis*	Porifera	659.87 ± 10.65	613.24 ± 10.76	11.70 ± 2.70	<5.00	<5.00
*Tripneustes ventricosus*	Echinodermata	11.03 ± 0.12	13.35 ± 3.91	25.00 ± 3.10	39.90 ± 2.00	77.00 ± 3.90
*Echinaster (Othilia) echinophorus*	Echinodermata	182.01 ± 67.12	112.55 ± 23.21	198.20 ± 27.20	265.70 ± 29.60	405.60 ± 50.40
*Isostichopus badionotus*	Echinodermata	1005.12 ± 293.32	11.08 ± 0.27	69.70 ± 10.00	57.10 ± 2.70	83.10 ± 3.00
*Stichodactyla helianthus*	Cnidaria	256.3 ± 10.00	136.56 ± 22.87	103.60 ± 20.60	110.80 ± 13.20	58.10 ± 7.50
*Bunodosoma granuliferum*	Cnidaria	ND	98.02 ± 18.05	567.60 ± 88.00	786.80 ± 37.10	711.30 ± 29.30
*Physalia physalis*	Cnidaria	ND	198.92 ± 10.76	123.10 ± 21.30	257.30 ± 6.70	234.50 ± 5.00
*Bestatin (positive control)*	-	25.50 ± 2.35	6.54 ± 0.82	6.70 ± 1.90	0.54 ± 0.01	3.15 ± 0.72
*Amastatin (positive control)*	-	75.45 ± 4.55	58.32 ± 3.34	63.45 ± 7.61	ND	ND

**Table 7:** Some members of the M17 family of the MF clan of metallopeptidases. (Compiled from https://www.ebi.ac.uk/merops/cgi-bin/, accessed on 11 January 2023).

Enzyme	IUBMB * Nomenclature	Merops ID	Sources
Leucyl aminopeptidase 3	3.4.11.1	M17.001	*Homo sapiens, Haemaphysalis longicornis, Mus musculus*
Leucyl aminopeptidase (plant-type)	3.4.11.1	M17.002	*Solanum lycopersicum*
PepA aminopeptidase	3.4.11.10	M17.003	*Escherichia coli*, *Pseudomonas aeruginosa*
PepB aminopeptidase	3.4.11.23	M17.004	*Escherichia coli*, *Salmonella typhimurium*
Mername-AA040 peptidase	-	M17.005	*Homo sapiens*
Leucyl aminopeptidase-1 (*Caenorhabditis*-type)	-	M17.006	*Caenorhabditis elegans*
M17 aminopeptidase (*Plasmodium* spp.)	3.4.11.1	M17.008	*Plasmodium* spp.
Aminopeptidase yspII (*Schizosaccharomyces* sp.)	-	M17.009	*Schizosaccharomyces* sp.
Leucyl aminopeptidase (*Bacillus*-type)	-	M17.010	*Geobacillus kaustophilus*
Leucyl aminopeptidase (*Fasciola*-type)	-	M17.011	*Fasciola hepática*
PwLAP aminopeptidase	-	M17.012	*Paragonimus westermani*
Cysteinylglycinase (*Treponema denticola*)-like peptidase	-	M17.013	*Treponema denticola*
LAPTc aminopeptidase	-	M17.014	*Trypanosoma cruzi, Leishmania major*
Aminopeptidase pepZ (*Staphylococcus* sp.)	-	M17.015	*Staphylococcus aureus*
Aminopeptidase A/I (*Helicobacter*-type)	-	M17.016	*Helicobacter pylori*
similar to cytosol aminopeptidase (*Rattus norvegicus*)	-	M17.950	*Rattus norvegicus*
At4g30920 (*Arabidopsis thaliana*)	-	M17.A01	*Arabidopsis thaliana*
At4g30910 (*Arabidopsis thaliana*)	-	M17.A02	*Arabidopsis thaliana*
At2g24200 (*Arabidopsis thaliana*)	-	M17.A03	*Arabidopsis thaliana*
CG7340 g.p. (*Drosophila melanogaster*)	-	M17.A04	*Drosophila melanogaster*
ZK353.6 (*Caenorhabditis elegans*)	-	M17.A05	*Caenorhabditis elegans*

* IUBMB: International Union of Biochemistry and Molecular Biology.

**Table 8:** Crystallographic structures reported for members of the M17 family of metallopeptidases (Compiled from https://www.ebi.ac.uk/merops/cgi-bin/, accessed on 11 January 2023).

Enzyme	Source	Crystallographic Codes of the Structures at Protein Data Bank (PDB)
Leucyl aminopeptidase 3	*Bos taurus*	1BLL, 1BPM, 1BPN, 1LAM, 1LAN, 1LAP, 1LCP, 2EWB, 2J9A
Leucyl aminopeptidase (plant-type)	*Solanum lycopersicum*	4KSI, 5D8N
PepA aminopeptidase	*Escherichia coli*	1GYT
	*Francisella tularensis*	3PEI
	*Pseudomonas putida*	3H8E, 3H8F, 3H8G
	*Xanthomonas oryzae*	3JRU
PepB aminopeptidase	*Escherichia coli*	6OV8
	*Yersinia pestis*	6CXD
M17 aminopeptidase (*Plasmodium* spp.)	*Plasmodium falciparum*	3KQX, 3KQZ, 3KR4, 3KR5, 3T8W, 4K3N, 4R6T, 4R7M, 4X2T
LAPTc aminopeptidase	*Leishmania major*	5NTH
	*Trypanosoma brucei*	5NSK, 5NSM, 5NSQ, 5NTD
Aminopeptidase A/I (*Helicobacter*-type)	*Helicobacter pylori*	4ZI6, 4ZLA
ZK353.6 (*Caenorhabditis elegans*)	*Caenorhabditis elegans*	2HB6, 2HC9

**Table 9:** Summary of the screening of inhibitory activity against malarial rPfA-M17 and hLAP in aqueous extracts from marine invertebrates from the Cuban coastline (see note below). (adapted from Pascual et al. [98]).

Species	Phyla	Crude Extract rPfA-M17 sIA (U/mg)	Crude Extract hLAP sIA (U/mg)	2.5% TCA Treated Extracts rPfA-M17 sIA (U/mg)	2.5% TCA Treated Extracts hLAP sIA (U/mg)
*Cenchritis muricatus*	Mollusca	0.40	0.16	7.56	4.30
*Nerita peloronta*	Mollusca	ND	ND	10.61	6.48
*Nerita versicolor*	Mollusca	ND	ND	7.17	5.58
*Lissodendoryx (Lissodendoryx) isodictyalis*	Porifera	ND	0.27	256.13	312.01
*Tripneustes ventricosus*	Equinodermata	ND	ND	41.85	43.31
*Echinaster (Othilia) echinophorus*	Equinodermata	0.11	ND	9.10	7.85
*Isostichopus badionotus*	Equinodermata	0.18	1.24	55.83	27.86
*Stichodactyla helianthus*	Cnidaria	0.55	0.68	38.15	18.67
*Bunodosoma granuliferum*	Cnidaria	0.19	ND	5.45	4.08
*Physalia physalis*	Cnidaria	0.40	ND	19.21	11.00

NOTE: Inhibitory activities found in aqueous crude and 2.5% TCA extracts are expressed as specific Inhibitory Activity (sIA) in U/mg. One unit of enzyme activity was defined as the amount of enzyme needed to produce one arbitrary unit of fluorescence (AUF) per minute, and inhibitory activities are expressed per mg of extracts. The first column indicates the species, and the remaining columns refer to specific sIA. (ND): not detected.

**Table 10:** Summary of the IC_50_ determination for the 2.5% TCA clarified extracts against rPfA-M17 and hLAP. Preliminarndicates position with high degree of conservation of the resy inhibitory analyses on the growth of *Plasmodium falciparum* FcB1 strain are included in the last column (adapted from Pascual et al. [98]).

Species	IC_50_ Value vs. rPfA-M17 (µg/mL)	IC_50_ vs. hLAP (µg/mL)	IC_50_hLAP/ IC_50_rPfA-M17	IC_50_ Pf FcB1 (µg/mL)
*Cenchritis muricatus*	113.40 ± 3.00	341.00 ± 110.00	3.00	>400
*Nerita peloronta*	22.20 ± 2.70	329.50 ± 100.00	14.80	291.80 ± 38.50
*Nerita versicolor*	207.00 ± 30.60	12 429.60 ± 633.00	60.00	325.50 ± 0.80
*Lissodendoryx (Lissodendoryx) isodictyalis*	27.30 ± 9.40	66.30 ± 27.60	2.42	2.60 ± 0.60
*Tripneustes ventricosus*	84.80 ± 7.30	607.70 ± 300.50	7.16	0.24 ± 0.01
*Echinaster (Othilia) echinophorus*	127.50 ± 82.10	308.70 ± 100.00	2.42	201.60 ± 162.10
*Isostichopus badionotus*	86.70 ± 32.60	272.70 ± 63.50	3.13	183.70 ± 155.70
*Stichodactyla helianthus*	15.30 ± 6.20	234.90 ± 34.60	15.35	>400
*Bunodosoma granuliferum*	509.20 ± 100.90	1171.00 ± 92.10	2.29	>400
*Physalia physalis*	293.70 ± 100.00	550.00 ± 85.00	1.87	206.00 ± 84.00
Bestatin (positive control)	0.15 ± 0.02	11.83 ± 2.61	78.86	1.14 ± 0.27
Amastatin (positive control)	60.70 ± 19.84	158.05 ± 28.44	2.60	ND

## Data Availability

Not applicable.

## References

[1] Rawlings N.D., Barrett A.J., Thomas P.D., Huang X., Bateman A., Finn R.D. (2018). The Merops Database of Proteolytic Enzymes, Their Substrates and Inhibitors in 2017 and a Comparison with Peptidases in the Panther Database. Nucleic Acids Res..

[2] Drag M., Salvesen G.S. (2010). Emerging Principles in Protease-Based Drug Discovery. Nat. Rev. Drug Discov..

[3] Deu E., Verdoes M., Bogyo M. (2012). New Approaches for Dissecting Protease Functions to Improve Probe Development and Drug Discovery. Nat. Struct. Mol. Biol..

[4] Kryvalap Y., Czyzyk J. (2022). The Role of Proteases and Serpin Protease Inhibitors in Β-Cell Biology and Diabetes. Biomolecules.

[5] Rai M., Curley M., Coleman Z., Demontis F. (2022). Contribution of Proteases to the Hallmarks of Aging and to Age-Related Neurodegeneration. Aging Cell.

[6] Verhulst E., Garnier D., De Meester I., Bauvois B. (2022). Validating Cell Surface Proteases as Drug Targets for Cancer Therapy: What Do We Know, and Where Do We Go?. Cancers.

[7] Newman D.J., Cragg G.M. (2016). Drugs and Drug Candidates from Marine Sources: An Assessment of the Current State of Play. Planta Med..

[8] Drinkwater N., Lee J., Yang W., Malcolm T.R., McGowan S. (2017). M1 Aminopeptidases as Drug Targets: Broad Applications or Therapeutic Niche?. FEBS J..

[9] Drinkwater N., Malcolm T.R., McGowan S. (2019). M17 Aminopeptidases Diversify Function by Moderating Their Macromolecular Assemblies and Active Site Environment. Biochimie.

[10] Hammers D., Carothers K., Lee S. (2022). The Role of Bacterial Proteases in Microbe and Host-Microbe Interactions. Curr. Drug Targets.

[11] Carvalho L.A., Bernardes G.J. (2022). The Impact of Activity-Based Protein Profiling in Malaria Drug Discovery. ChemMedChem.

[12] Beltran-Hortelano I., Alcolea V., Font M., Pérez-Silanes S. (2022). Examination of Multiple *Trypanosoma cruzi* Targets in a New Drug Discovery Approach for Chagas Disease. Bioorganic Med. Chem..

[13] Vermelho A.B., Cardoso V., Mansoldo F.R.P., Supuran C.T., Cedrola S.M.L., Rodrigues I.A., Rodrigues G.C. (2022). Chagas Disease: Drug Development and Parasite Targets. Antiprotozoal Drug Development and Delivery.

[14] Mukherjee R., Dikic I. (2022). Regulation of Host-Pathogen Interactions Via the Ubiquitin System. Annu. Rev. Microbiol..

[15] Salomon E., Schmitt M., Marapaka A.K., Stamogiannos A., Revelant G., Schmitt C., Alavi S., Florent I., Addlagatta A., Stratikos E. (2018). Aminobenzosuberone Scaffold as a Modular Chemical Tool for the Inhibition of Therapeutically Relevant M1 Aminopeptidases. Molecules.

[16] Albiston A.L., Ye S., Chai S.Y. (2004). Membrane Bound Members of the M1 Family: More Than Aminopeptidases. Protein Pept. Lett..

[17] Carl-McGrath S., Lendeckel U., Ebert M., Röcken C. (2006). Ectopeptidases in Tumour Biology: A Review. Histol. Histopathol..

[18] Mucha A., Drag M., Dalton J.P., Kafarski P. (2010). Metallo-Aminopeptidase Inhibitors. Biochimie.

[19] Amin S.A., Adhikari N., Jha T. (2018). Design of Aminopeptidase N Inhibitors as Anti-Cancer Agents. J. Med. Chem..

[20] Alvarez C., Pazos F., Soto C., Laborde R., Lanio M.E. (2020). Pore-Forming Toxins from Sea Anemones: From Protein-Membrane Interaction to Its Implications for Developing Biomedical Applications. Advances in Biomembranes and Lipid Self-Assembly.

[21] Alvarez C., Soto C., Cabezas S., Alvarado-Mesén J., Laborde R., Pazos F., Ros U., Hernández A.M., Lanio M.E. (2021). Panorama of the Intracellular Molecular Concert Orchestrated by Actinoporins, Pore-Forming Toxins from Sea Anemones. Toxins.

[22] Martell E.M., González-Garcia M., Ständker L., Otero-González A.J. (2021). Host Defense Peptides as Immunomodulators: The Other Side of the Coin. Peptides.

[23] Rodríguez A.A., Otero-González A., Ghattas M., Ständker L. (2021). Discovery, Optimization, and Clinical Application of Natural Antimicrobial Peptides. Biomedicines.

[24] Riccio G., Ruocco N., Mutalipassi M., Costantini M., Zupo V., Coppola D., De Pascale D., Lauritano C. (2020). Ten-Year Research Update Review: Antiviral Activities from Marine Organisms. Biomolecules.

[25] Nakao Y., Fusetani N. (2007). Enzyme Inhibitors from Marine Invertebrates. J. Nat. Prod..

[26] Pujiastuti D.Y., Amin M.N.G., Alamsjah M.A., Hsu J.-L. (2019). Marine Organisms as Potential Sources of Bioactive Peptides That Inhibit the Activity of Angiotensin I-Converting Enzyme: A Review. Molecules.

[27] Tischler D. (2020). A Perspective on Enzyme Inhibitors from Marine Organisms. Mar. Drugs.

[28] Moodie L.W.K., Sepčić K., Turk T., Frangež R., Svenson J. (2019). Natural Cholinesterase Inhibitors from Marine Organisms. Nat. Prod. Rep..

[29] Alonso-del-Rivero M., Trejo S.A., Rodriguez de la Vega M., González Y., Bronsoms S., Canals F., Delfín J., Diaz J., Aviles F.X., Chávez M.A. (2009). A Novel Metallocarboxypeptidase-Like Enzyme from the Marine Annelid *Sabellastarte magnifica*–a Step into the Invertebrate World of Proteases. FEBS J..

[30] Alonso I.P., Méndez L.R., Almeida F., Tresano M.E.V., Sánchez Y.A., Hernández-Zanuy A., Álvarez-Lajonchere L., Díaz D., Sánchez B., Florent I. (2020). Marine Organisms: A Source of Biomedically Relevant Metallo M1, M2 and M17 Exopeptidase Inhibitors. Rev. Cuba. Cienc. Biológicas.

[31] Chávez M., Delfín J., Díaz J., Pérez U., Martínez J., González J., Márquez M., Más R. (1988). Caracterización de un Inhibidor de Proteasas Obtenido de la Anémona *S. helianthus*. Rev. CENIC.

[32] Delfin J., Gonzalez Y., Diaz J., Chavez M. (1994). Proteinase Inhibitor from *Stichodactyla helianthus*: Purification, Characterization and Immobilization. Arch. Med. Res..

[33] Delfin J., Martinez I., Antuch W., Morera V., Gonzalez Y., Rodriguez R., Marquez M., Saroyan A., Larionova N., Diaz J. (1996). Purification, Characterization and Immobilization of Proteinase Inhibitors from *Stichodactyla helianthus*. Toxicon.

[34] Lenarčič B., Ritonja A., Štrukelj B., Turk B., Turk V. (1997). Equistatin, a New Inhibitor of Cysteine Proteinases from *Actinia equina*, Is Structurally Related to Thyroglobulin Type-1 Domain. J. Biol. Chem..

[35] González Y., Araujo M., Oliva M., Sampaio C., Chávez M. (2004). Purification and Preliminary Characterization of a Plasma Kallikrein Inhibitor Isolated from Sea Hares *Aplysia dactylomela* Rang, 1828. Toxicon.

[36] Reytor M.L., González Y., Pascual I., Hernández A., Chávez M Á., Alonso del Rivero M. (2011). Screening of Protease Inhibitory Activity in Extracts of Five Ascidian Species from Cuban Coasts. Biotecnol. Apl..

[37] Alonso-Del-Rivero M., Trejo S., Reytor M.L., Rodriguez-De-La-Vega M., Delfin J., Diaz J., Gonzalez M.L.R., Canals F., Chavez M.A., Aviles F.X. (2012). Tri-Domain Bifunctional Inhibitor of Metallocarboxypeptidases a and Serine Proteases Isolated from Marine Annelid *Sabellastarte magnifica*. J. Biol. Chem..

[38] Salas-Sarduy E., Cabrera-Muñoz A., Cauerhff A., González-González Y., Trejo S.A., Chidichimo A., de los Angeles Chávez-Planes M., José Cazzulo J. (2013). Antiparasitic Effect of a Fraction Enriched in Tight-Binding Protease Inhibitors Isolated from the Caribbean Coral *Plexaura homomalla*. Exp. Parasitol..

[39] González L., Sánchez R.E., Rojas L., Pascual I., García-Fernández R., Chávez M.A., Betzel C. (2016). Screening of Protease Inhibitory Activity in Aqueous Extracts of Marine Invertebrates from Cuban Coast. Am. J. Anal. Chem..

[40] Salas-Sarduy E., Guerra Y., Covaleda Cortés G., Avilés F.X., Chávez Planes M.A. (2017). Identification of Tight-Binding Plasmepsin Ii and Falcipain 2 Inhibitors in Aqueous Extracts of Marine Invertebrates by the Combination of Enzymatic and Interaction-Based Assays. Mar. Drugs.

[41] Covaleda G., Trejo S.A., Salas-Sarduy E., del Rivero M.A., Chavez M.A., Aviles F.X. (2017). Intensity Fading Maldi-Tof Mass Spectrometry and Functional Proteomics Assignments to Identify Protease Inhibitors in Marine Invertebrates. J. Proteom..

[42] Hong T.T., Dat T.T.H., Cuong P.V., Cuc N.T.K. (2018). Protease Inhibitors from Marine Sponge and Sponge-Associated Microorganisms. Vietnam. J. Sci. Technol..

[43] Ikegami S., Kobayashi H., Myotoishi Y., Ohta S., Kato K.H. (1994). Selective Inhibition of Exoplasmic Membrane Fusion in Echinoderm Gametes with Jaspisin, a Novel Antihatching Substance Isolated from a Marine Sponge. J. Biol. Chem..

[44] Kato K.H., Takemoto K., Kato E., Miyazaki K., Kobayashi H., Ikegami S. (1998). Inhibition of Sea Urchin Fertilization by Jaspisin, a Specific Inhibitor of Matrix Metalloendoproteinase. Dev. Growth Differ..

[45] Fusetani N., Fujita M., Nakao Y., Matsunaga S., van Soest R.W. (1999). Tokaramide a, a New Cathepsin B Inhibitor from the Marine Sponge *Theonella Aff. mirabilis*. Bioorganic Med. Chem. Lett..

[46] Fujita M., Nakao Y., Matsunaga S., van Soest R.W., Itoh Y., Seiki M., Fusetani N. (2003). Callysponginol Sulfate a, an Mt1-Mmp Inhibitor Isolated from the Marine Sponge *Callyspongia tuncata*. J. Nat. Prod..

[47] Fujita M., Nakao Y., Matsunaga S., Seiki M., Itoh Y., Yamashita J., Van Soest R.W., Fusetani N. (2003). Ageladine A: An Antiangiogenic Matrixmetalloproteinase Inhibitor from the Marine Sponge *Agelas N Akamurai*. J. Am. Chem. Soc..

[48] Shim J.S., Lee H.-S., Shin J., Kwon H.J. (2004). Psammaplin a, a Marine Natural Product, Inhibits Aminopeptidase N and Suppresses Angiogenesis in Vitro. Cancer Lett..

[49] Pascual I., Gil-Parrado S., Cisneros M., Joseph-Bravo P., Dıaz J., Possani L.D., Charli J.L., Chávez M. (2004). Purification of a Specific Inhibitor of Pyroglutamyl Aminopeptidase Ii from the Marine Annelide *Hermodice carunculata*: In Vivo Effects in Rodent Brain. Int. J. Biochem. Cell Biol..

[50] Thomas N.V., Kim S.-K. (2010). Metalloproteinase Inhibitors: Status and Scope from Marine Organisms. Biochem. Res. Int..

[51] del Rivero M.A., Reytor M.L., Trejo S.A., Chávez M.A., Avilés F.X., Reverter D. (2013). A Noncanonical Mechanism of Carboxypeptidase Inhibition Revealed by the Crystal Structure of the Tri-Kunitz Smci in Complex with Human Cpa4. Structure.

[52] Covaleda G., del Rivero M.A., Chávez M.A., Avilés F.X., Reverter D. (2012). Crystal Structure of Novel Metallocarboxypeptidase Inhibitor from Marine Mollusk *Nerita versicolor* in Complex with Human Carboxypeptidase A4. J. Biol. Chem..

[53] Cabrera-Muñoz A., Valiente P.A., Rojas L., Antigua M.A.D.R., Pires J.R. (2019). Nmr Structure of Cmpi-Ii, a Non-Classical Kazal Protease Inhibitor: Understanding Its Conformational Dynamics and Subtilisin a Inhibition. J. Struct. Biol..

[54] Pascual Alonso I., Rivera Méndez L., Valdés-Tresanco M.E., Bounaadja L., Schmitt M., Arrebola Sánchez Y., Alvarez Lajonchere L., Charli J.L., Florent I. (2020). Biochemical Evidences for M1-, M17- and M18-Like Aminopeptidases in Marine Invertebrates from Cuban Coastline. Z. Für Nat. C.

[55] Sarfaraj H.M., Sheeba F., Saba A., Khan M. (2012). Marine Natural Products: A Lead for Anti-Cancer.

[56] Nakao Y., Oku N., Matsunaga S., Fusetani N. (1998). Cyclotheonamides E2 and E3, New Potent Serine Protease Inhibitors from the Marine Sponge of the Genus *Theonella*. J. Nat. Prod..

[57] Hanessian S., Tremblay M., Petersen J.F. (2004). The N-Acyloxyiminium Ion Aza-Prins Route to Octahydroindoles: Total Synthesis and Structural Confirmation of the Antithrombotic Marine Natural Product Oscillarin. J. Am. Chem. Soc..

[58] Gunasekera S.P., McCarthy P.J., Longley R.E., Pomponi S.A., Wright A.E., Lobkovsky E., Clardy J. (1999). Discorhabdin P, a New Enzyme Inhibitor from a Deep-Water Caribbean Sponge of the Genus *Batzella*. J. Nat. Prod..

[59] Gunasekera S.P., McCarthy P.J., Longley R.E., Pomponi S.A., Wright A.E. (1999). Secobatzellines a and B, Two New Enzyme Inhibitors from a Deep-Water Caribbean Sponge of the Genus *Batzella*. J. Nat. Prod..

[60] Hu J.F., Schetz J.A., Kelly M., Peng J.N., Ang K.K., Flotow H., Leong C.Y., Ng S.B., Buss A.D., Wilkins S.P. (2002). New Antiinfective and Human 5-Ht2 Receptor Binding Natural and Semisynthetic Compounds from the Jamaican Sponge *Smenospongia aurea*. J. Nat. Prod..

[61] Fujita M., Nakao Y., Matsunaga S., Nishikawa T., Fusetani N. (2002). Sodium 1-(12-Hydroxy) Octadecanyl Sulfate, an Mmp2 Inhibitor, Isolated from a Tunicate of the Family *Polyclinidae*. J. Nat. Prod..

[62] Joe M.-J., Kim S.-N., Choi H.-Y., Shin W.-S., Park G.-M., Kang D.-W., Kim Y.K. (2006). The Inhibitory Effects of Eckol and Dieckol from *Ecklonia stolonifera* on the Expression of Matrix Metalloproteinase-1 in Human Dermal Fibroblasts. Biol. Pharm. Bull..

[63] Harper J.W., Powers J.C. (1986). Inhibitors of Metallo-Proteases. Proteinase Inhibitors.

[64] Rawlings N.D., Barrett A.J. (1993). Evolutionary Families of Peptidases. Biochem. J..

[65] Rawlings N.D., Barrett A.J. (1995). [13] Evolutionary Families of Metallopeptidases. Methods in Enzymology.

[66] Reeck G.R., de Haën C., Teller D.C., Doolittle R.F., Fitch W.M., Dickerson R.E., Chambon P., McLachlan A.D., Margoliash E., Jukes T.H. (1987). Homology in Proteins and Nucleic Acids: A Terminology Muddle and a Way out of It. Cell.

[67] Haeggström J.Z., Nordlund P., Thunnissen M.M. (2002). Functional Properties and Molecular Architecture of Leukotriene A4 Hydrolase, a Pivotal Catalyst of Chemotactic Leukotriene Formation. Sci. World J..

[68] Fukasawa K.M., Fukasawa K., Harada M., Hirose J., Izumi T., Shimizu T. (1999). Aminopeptidase B Is Structurally Related to Leukotriene-A4 Hydrolase but Is Not a Bifunctional Enzyme with Epoxide Hydrolase Activity. Biochem. J..

[69] Klinke T., Rump A., Pönisch R., Schellenberger W., Müller E.C., Otto A., Klimm W., Kriegel T.M. (2008). Identification and Characterization of Caape2–a Neutral Arginine/Alanine/Leucine-Specific Metallo-Aminopeptidase from *Candida albicans*. FEMS Yeast Res..

[70] Wong A.H.M., Zhou D., Rini J.M. (2012). The X-Ray Crystal Structure of Human Aminopeptidase N Reveals a Novel Dimer and the Basis for Peptide Processing. J. Biol. Chem..

[71] Thunnissen M.M., Nordlund P., Haeggström J.Z. (2001). Crystal Structure of Human Leukotriene A4 Hydrolase, a Bifunctional Enzyme in Inflammation. Nat. Struct. Biol..

[72] Kyrieleis O.J., Goettig P., Kiefersauer R., Huber R., Brandstetter H. (2005). Crystal Structures of the Tricorn Interacting Factor F3 from Thermoplasma Acidophilum, a Zinc Aminopeptidase in Three Different Conformations. J. Mol. Biol..

[73] Ito K., Nakajima Y., Onohara Y., Takeo M., Nakashima K., Matsubara F., Ito T., Yoshimoto T. (2006). Crystal Structure of Aminopeptidase N (Proteobacteria Alanyl Aminopeptidase) from *Escherichia coli* and Conformational Change of Methionine 260 Involved in Substrate Recognition. J. Biol. Chem..

[74] McGowan S., Porter C.J., Lowther J., Stack C.M., Golding S.J., Skinner-Adams T.S., Trenholme K.R., Teuscher F., Donnelly S.M., Grembecka J. (2009). Structural Basis for the Inhibition of the Essential Plasmodium Falciparum M1 Neutral Aminopeptidase. Proc. Natl. Acad. Sci. USA.

[75] Harbut M.B., Velmourougane G., Dalal S., Reiss G., Whisstock J.C., Onder O., Brisson D., McGowan S., Klemba M., Greenbaum D.C. (2011). Bestatin-Based Chemical Biology Strategy Reveals Distinct Roles for Malaria M1-and M17-Family Aminopeptidases. Proc. Natl. Acad. Sci. USA.

[76] Salomon E., Schmitt M., Mouray E., McEwen A.G., Bounaadja L., Torchy M., Poussin-Courmontagne P., Alavi S., Tarnus C., Cavarelli J. (2020). Aminobenzosuberone Derivatives as Pfa-M1 Inhibitors: Molecular Recognition and Antiplasmodial Evaluation. Bioorganic Chem..

[77] Yang Y., Liu C., Lin Y.-L., Li F. (2013). Structural Insights into Central Hypertension Regulation by Human Aminopeptidase A. J. Biol. Chem..

[78] Kochan G., Krojer T., Harvey D., Fischer R., Chen L., Vollmar M., von Delft F., Kavanagh K.L., Brown M.A., Bowness P. (2011). Crystal Structures of the Endoplasmic Reticulum Aminopeptidase-1 (Erap1) Reveal the Molecular Basis for N-Terminal Peptide Trimming. Proc. Natl. Acad. Sci. USA.

[79] Chen J., Wang Y., Zhong Q., Wu Y., Xia W. (2012). Purification and Characterization of a Novel Angiotensin-I Converting Enzyme (Ace) Inhibitory Peptide Derived from Enzymatic Hydrolysate of Grass Carp Protein. Peptides.

[80] Gago F. (2023). Computational Approaches to Enzyme Inhibition by Marine Natural Products in the Search for New Drugs. Mar. Drugs.

[81] Omar A.M., Mohammad K.A., Sindi I.A., Mohamed G.A., Ibrahim S.R.M. (2023). Unveiling the Efficacy of Sesquiterpenes from Marine Sponge *Dactylospongia elegans* in Inhibiting Dihydrofolate Reductase Using Docking and Molecular Dynamic Studies. Molecules.

[82] Joseph-Bravo P., Jaimes-Hoy L., Uribe R.M., Charli J.L. (2015). 60 Years of Neuroendocrinology: TRH, the First Hypophysiotropic Releasing Hormone Isolated: Control of the Pituitary–Thyroid Axis. J. Endocrinol..

[83] Kronenberg H.M., Shlomo Melmed M.D., Polonsky K.S., Wilson J.D., Foster D.W., Kronenberg H.M. (2002). Williams Textbook of Endocrinology.

[84] Charli J.L., Rodríguez-Rodríguez A., Hernández-Ortega K., Cote-Vélez A., Uribe R.M., Jaimes-Hoy L., Joseph-Bravo P. (2020). The Thyrotropin-Releasing Hormone-Degrading Ectoenzyme, a Therapeutic Target?. Front. Pharm..

[85] A Kelly J. (1995). Thyrotropin-Releasing Hormone: Basis and Potential for Its Therapeutic Use. Essays Biochem..

[86] Charli J.-L., Mendez M., Vargas M.-A., Cisneros M., Assai M., Joseph-Bravo P., Wilk S. (1989). Pyroglutamyl Peptidase Ii Inhibition Specifically Increases Recovery of Trh Released from Rat Brain Slices. Neuropeptides.

[87] Kelly J.A., Slator G.R., Tipton K.F., Williams C.H., Bauer K. (2000). Kinetic Investigation of the Specificity of Porcine Brain Thyrotropin-Releasing Hormone-Degrading Ectoenzyme for Thyrotropin-Releasing Hormone-Like Peptides. J. Biol. Chem..

[88] Kelly J.A., Scalabrino G.A., Slator G.R., Cullen A.A., Gilmer J.F., Lloyd D.G., Bennett G.W., Bauer K., Tipton K.F., Williams C.H. (2005). Structure–Activity Studies with High-Affinity Inhibitors of Pyroglutamyl-Peptidase Ii. Biochem. J..

[89] Cruz R., Vargas M.A., Uribe R.M., Pascual I., Lazcano I., Yiotakis A., Matziari M., Joseph-Bravo P., Charli J.-L. (2008). Anterior Pituitary Pyroglutamyl Peptidase Ii Activity Controls Trh-Induced Prolactin Release. Peptides.

[90] Sinko R., Mohácsik P., Kővári D., Penksza V., Wittmann G., Mácsai L., Fonseca T.L., Bianco A.C., Fekete C., Gereben B. (2023). Different Hypothalamic Mechanisms Control Decreased Circulating Thyroid Hormone Levels in Infection and Fasting-Induced Non-Thyroidal Illness Syndrome in Male Thyroid Hormone Action Indicator Mice. Thyroid.

[91] Barnieh F.M., Loadman P.M., Falconer R.A. (2021). Is Tumour-Expressed Aminopeptidase N (Apn/Cd13) Structurally and Functionally Unique?. Biochim. Biophys. Acta (BBA)-Rev. Cancer.

[92] Wickström M., Larsson R., Nygren P., Gullbo J. (2011). Aminopeptidase N (Cd13) as a Target for Cancer Chemotherapy. Cancer Sci..

[93] Ni J., Wang X., Shang Y., Li Y., Chen S. (2021). Cd13 Inhibition Augments Dr4-Induced Tumor Cell Death in a P-Erk1/2-Independent Manner. Cancer Biol. Med..

[94] Schreiter A., Gore C., Labuz D., Fournie-Zaluski M.C., Roques B.P., Stein C., Machelska H. (2012). Pain Inhibition by Blocking Leukocytic and Neuronal Opioid Peptidases in Peripheral Inflamed Tissue. FASEB J..

[95] Bonnard E., Poras H., Nadal X., Maldonado R., Fournié-Zaluski M.C., Roques B.P. (2015). Long-Lasting Oral Analgesic Effects of N-Protected Aminophosphinic Dual Enk Ephalinase Inhibitors (Denki S) in Peripherally Controlled Pain. Pharmacol. Res. Perspect..

[96] Arrebola Y., Rivera L., Pedroso A., McGuire R., Tresanco M.E.V., Bergado G., Charli J.-L., Sánchez B., Alonso I.P. (2021). Bacitracin Is a Non-Competitive Inhibitor of Porcine M1 Family Neutral and Glutamyl Aminopeptidases. Nat. Prod. Res..

[97] Melzig M.F., Bormann H. (1998). Betulinic Acid Inhibits Aminopeptidase N Activity. Planta Med..

[98] Pascual Alonso I., Bounaadja L., Sánchez L., Rivera L., Tarnus C., Schmitt M., Garcia G., Diaz L., Hernandez-Zanuy A., Sánchez B. (2017). Aqueous Extracts of Marine Invertebrates from Cuba Coastline Display Neutral Aminopeptidase Inhibitory Activities and Effects on Cancer Cells and Plasmodium Falciparum Parasites. Indian J. Nat. Prod. Resour..

[99] Pascual I., Valiente P.A., García G., Valdés-Tresanco M.E., Arrebola Y., Díaz L., Bounaadja L., Uribe R.M., Pacheco M.C., Florent I. (2017). Discovery of Novel Non-Competitive Inhibitors of Mammalian Neutral M1 Aminopeptidase (Apn). Biochimie.

[100] Pascual-Alonso I., Alonso-Bosch R., Cabrera-Muñoz A., Perera W.H., Charli J.L. (2019). Methanolic Extracts of Paratoid Gland Secretions from Cuban *Peltophryne* Toads Contain Inhibitory Activities against Peptidases with Biomedical Relevance. Biotecnol. Apl..

[101] Alonso I.P., Méndez L.R., García F.A., Valdés-Tresanco M.E., Bosch R.A., Perera W.H., Sánchez Y.A., Bergado G., Ramírez B.S., Charli J.-L. (2023). Bufadienolides Preferentially Inhibit Aminopeptidase N among Mammalian Metallo-Aminopeptidases; Relationship with Effects on Human Melanoma Mewo Cells. Int. J. Biol. Macromol..

[102] Wang E., Sorolla M.A., Krishnan P.D.G., Sorolla A. (2020). From Seabed to Bedside: A Review on Promising Marine Anticancer Compounds. Biomolecules.

[103] Malla R.R., Farran B., Nagaraju G.P. (2021). Understanding the Function of the Tumor Microenvironment, and Compounds from Marine Organisms for Breast Cancer Therapy. World J. Biol. Chem..

[104] Aldrich L.N., Burdette J.E., de Blanco E.C., Coss C.C., Eustaquio A.S., Fuchs J.R., Kinghorn A.D., MacFarlane A., Mize B.K., Oberlies N.H. (2022). Discovery of Anticancer Agents of Diverse Natural Origin. J. Nat. Prod..

[105] Nuzzo G., Senese G., Gallo C., Albiani F., Romano L., D’ippolito G., Manzo E., Fontana A. (2022). Antitumor Potential of Immunomodulatory Natural Products. Mar. Drugs.

[106] Saeed A.F., Su J., Ouyang S. (2021). Marine-Derived Drugs: Recent Advances in Cancer Therapy and Immune Signaling. Biomed. Pharmacother..

[107] Jung J.H., Sim C.J., Lee C.-O. (1995). Cytotoxic Compounds from a Two-Sponge Association. J. Nat. Prod..

[108] Shin J., Lee H.-S., Seo Y., Rho J.-R., Cho K.W., Paul V.J. (2000). New Bromotyrosine Metabolites from the Sponge *Aplysinella rhax*. Tetrahedron.

[109] Pina I.C., Gautschi J.T., Wang G.Y.S., Sanders M.L., Schmitz F.J., France D., Cornell-Kennon S., Sambucetti L.C., Remiszewski S.W., Perez L.B. (2003). Psammaplins from the Sponge Pseudoceratina P Urpurea: Inhibition of Both Histone Deacetylase and DNA Methyltransferase. J. Org. Chem..

[110] Park Y., Liu Y., Hong J., Lee C.O., Cho H., Kim D.K., Im K.S., Jung J.H. (2003). New Bromotyrosine Derivatives from an Association of Two Sponges, Jaspis W Ondoensis and Poecillastra W Ondoensis. J. Nat. Prod..

[111] Yang Q., Liu D., Sun D., Yang S., Hu G., Wu Z., Zhao L. (2010). Synthesis of the Marine Bromotyrosine Psammaplin F and Crystal Structure of a Psammaplin a Analogue. Molecules.

[112] Mujumdar P., Teruya K., Tonissen K.F., Vullo D., Supuran C.T., Peat T.S., Poulsen S.A. (2016). An Unusual Natural Product Primary Sulfonamide: Synthesis, Carbonic Anhydrase Inhibition, and Protein X-Ray Structures of Psammaplin C. J. Med. Chem..

[113] Kim D., Lee I.S., Jung J.H., Yang S.I. (1999). Psammaplin a, a Natural Bromotyrosine Derivative from a Sponge, Possesses the Antibacterial Activity against Methicillin-Resistant *Staphylococcus aureus* and the DNA Gyrase-Inhibitory Activity. Arch. Pharmacal Res..

[114] TTabudravu J., Eijsink V., Gooday G., Jaspars M., Komander D., Legg M., Synstad B., van Aalten D. (2002). Psammaplin a, a Chitinase Inhibitor Isolated from the Fijian Marine Sponge *Aplysinella rhax*. Bioorganic Med. Chem..

[115] Revelant G., Al-Lakkis-Wehbe M., Schmitt M., Alavi S., Schmitt C., Roux L., Al-Masri M., Schifano-Faux N., Maiereanu C., Tarnus C. (2015). Exploring S1 Plasticity and Probing S1′ Subsite of Mammalian Aminopeptidase N/Cd13 with Highly Potent and Selective Aminobenzosuberone Inhibitors. Bioorganic Med. Chem..

[116] Kim T.H., Kim H.S., Kang Y.J., Yoon S., Lee J., Choi W.S., Jung J.H., Kim H.S. (2015). Psammaplin a Induces Sirtuin 1-Dependent Autophagic Cell Death in Doxorubicin-Resistant Mcf-7/Adr Human Breast Cancer Cells and Xenografts. Biochim. Biophys. Acta (BBA)-Gen. Subj..

[117] Ratovitski E.A. (2016). Tumor Protein (Tp)-P53 Members as Regulators of Autophagy in Tumor Cells Upon Marine Drug Exposure. Mar. Drugs.

[118] Zhou Y.-D., Li J., Du L., Mahdi F., Le T.P., Chen W.-L., Swanson S.M., Watabe K., Nagle D.G. (2018). Biochemical and Anti-Triple Negative Metastatic Breast Tumor Cell Properties of Psammaplins. Mar. Drugs.

[119] Ahn M.Y., Jung J.H., Na Y.J., Kim H.S. (2008). A Natural Histone Deacetylase Inhibitor, Psammaplin a, Induces Cell Cycle Arrest and Apoptosis in Human Endometrial Cancer Cells. Gynecol. Oncol..

[120] Kim H.J., Kim J.H., Chie E.K., Da Young P., Kim I.A., Kim I.H. (2012). Dnmt (DNA Methyltransferase) Inhibitors Radiosensitize Human Cancer Cells by Suppressing DNA Repair Activity. Radiat. Oncol..

[121] Kim D.H., Shin J., Kwon H.J. (2007). Psammaplin a Is a Natural Prodrug That Inhibits Class I Histone Deacetylase. Exp. Mol. Med..

[122] Jing Q., Hu X., Ma Y., Mu J., Liu W., Xu F., Li Z., Bai J., Hua H., Li D. (2019). Marine-Derived Natural Lead Compound Disulfide-Linked Dimer Psammaplin A: Biological Activity and Structural Modification. Mar. Drugs.

[123] Nicolaou K.C., Hughes R., Pfefferkorn J.A., Barluenga S., Roecker A.J. (2001). Combinatorial Synthesis through Disulfide Exchange: Discovery of Potent Psammaplin a Type Antibacterial Agents Active against Methicillin-Resistant *Staphylococcus aureus* (MRSA). Chem. –A Eur. J..

[124] Nicolaou K.C., Hughes R., Pfefferkorn J.A., Barluenga S. (2001). Optimization and Mechanistic Studies of Psammaplin a Type Antibacterial Agents Active against Methicillin-Resistant *Staphylococcus aureus* (MRSA). Chem. –A Eur. J..

[125] Alonso I.P., Sanchez Y.M.A., Ruiz G.A., González M.L.R., Hernández-Zanuy A. (2016). Identificación De Actividad Inhibidora De Aminopeptidasa N Aislada De Riñón Porcino, En Invertebrados Marinos De La Plataforma Insular De La Habana/Identification of Porcine Kidney Aminopeptidase N Inhibitory Activity, in Marine Invertebrates from Havana Coastline. Rev. Cuba. Cienc. Biológicas.

[126] Alonso I.P., Pedroso A., Sánchez Y.M.A., Valdés-Tresanco M.E., Méndez L.R., Fortun S. (2018). La Aminopeptidasa a De Mamíferos: Características Bioquímicas, Funciones Fisiológicas Y Su Implicación En Procesos Fisiopatológicos En Humanos/Aminopeptidase a from Mammals: Biochemical Characteristics, Physiological Roles and Implication in Physiopathological Processes in Humans. Rev. Cuba. Cienc. Biológicas.

[127] Göhring B., Holzhausen H., Meye A., Heynemann H., Rebmann U., Langner J., Riemann D. (1998). Endopeptidase 24.11/Cd10 Is Down-Regulated in Renal Cell Cancer. Int. J. Mol. Med..

[128] Tonna S., Dandapani S.V., Uscinski A., Appel G.B., Schlöndorff J.S., Zhang K., Denker B.M., Pollak M.R. (2008). Functional Genetic Variation in Aminopeptidase a (Enpep): Lack of Clear Association with Focal and Segmental Glomerulosclerosis (Fsgs). Gene.

[129] del Carmen Puertas M., Martínez-Martos J.M., Cobo M., Lorite P., Sandalio R.M., Palomeque T., Torres M.I., Carrera-González M.P., Mayas M.D., Ramírez-Expósito M.J. (2013). Plasma Renin–Angiotensin System-Regulating Aminopeptidase Activities Are Modified in Early Stage Alzheimer’s Disease and Show Gender Differences but Are Not Related to Apolipoprotein E Genotype. Exp. Gerontol..

[130] Blanco L., Sanz B., Perez I., Sánchez C.E., Cándenas M.L., Pinto F.M., Gil J., Casis L., López J.I., Larrinaga G. (2014). Altered Glutamyl-Aminopeptidase Activity and Expression in Renal Neoplasms. BMC Cancer.

[131] Bodineau L., Frugiere A., Marc Y., Inguimbert N., Fassot C., Balavoine F., Roques B., Llorens-Cortes C. (2008). Orally Active Aminopeptidase a Inhibitors Reduce Blood Pressure: A New Strategy for Treating Hypertension. Hypertension.

[132] Matsui M., Fowler J.H., Walling L.L. (2006). Leucine Aminopeptidases: Diversity in Structure and Function. Biol. Chem..

[133] Scranton M.A., Yee A., Park S.-Y., Walling L.L. (2012). Plant Leucine Aminopeptidases Moonlight as Molecular Chaperones to Alleviate Stress-Induced Damage. J. Biol. Chem..

[134] Chao W.S., Gu Y.-Q., Pautot V., Bray E.A., Walling L.L. (1999). Leucine Aminopeptidase Rnas, Proteins, and Activities Increase in Response to Water Deficit, Salinity, and the Wound Signals Systemin, Methyl Jasmonate, and Abscisic Acid. Plant Physiol..

[135] Alén C., Sherratt D.J., Colloms S. (1997). Direct Interaction of Aminopeptidase a with Recombination Site DNA in Xer Site-Specific Recombination. EMBO J..

[136] Behari J., Stagon L., Calderwood S.B. (2001). Pepa, a Gene Mediating Ph Regulation of Virulence Genes in *Vibrio cholerae*. J. Bacteriol..

[137] Aly A.S., Vaughan A.M., Kappe S.H. (2009). Malaria Parasite Development in the Mosquito and Infection of the Mammalian Host. Annu. Rev. Microbiol..

[138] Goldberg D.E. (2013). Complex Nature of Malaria Parasite Hemoglobin Degradation. Proc. Natl. Acad. Sci. USA.

[139] Skinner-Adams T.S., Stack C.M., Trenholme K.R., Brown C.L., Grembecka J., Lowther J., Mucha A., Drag M., Kafarski P., McGowan S. (2010). *Plasmodium falciparum* Neutral Aminopeptidases: New Targets for Anti-Malarials. Trends Biochem. Sci..

[140] Calic P.P., Vinh N.B., Webb C.T., Malcolm T.R., Ngo A., Lowes K., Drinkwater N., McGowan S., Scammells P.J. (2023). Structure-Based Development of Potent Plasmodium Falciparum M1 and M17 Aminopeptidase Selective and Dual Inhibitors Via S1’-Region Optimisation. Eur. J. Med. Chem..

[141] Drinkwater N., Vinh N.B., Mistry S.N., Bamert R.S., Ruggeri C., Holleran J.P., Loganathan S., Paiardini A., Charman S.A., Powell A.K. (2016). Potent Dual Inhibitors of *Plasmodium falciparum* M1 and M17 Aminopeptidases through Optimization of S1 Pocket Interactions. Eur. J. Med. Chem..

[142] Bauvois B., Dauzonne D. (2006). Aminopeptidase-N/Cd13 (Ec 3.4. 11.2) Inhibitors: Chemistry, Biological Evaluations, and Therapeutic Prospects. Med. Res. Rev..

[143] Kancharla P., Li Y., Yeluguri M., Dodean R.A., Reynolds K.A., Kelly J.X. (2021). Total Synthesis and Antimalarial Activity of 2-(P-Hydroxybenzyl)-Prodigiosins, Isoheptylprodigiosin, and Geometric Isomers of Tambjamine Myp1 Isolated from Marine Bacteria. J. Med. Chem..

[144] Nweze J.A., Mbaoji F.N., Li Y.-M., Yang L.-Y., Huang S.-S., Chigor V.N., Eze E.A., Pan L.-X., Zhang T., Yang D.-F. (2021). Potentials of Marine Natural Products against Malaria, Leishmaniasis, and Trypanosomiasis Parasites: A Review of Recent Articles. Infect. Dis. Poverty.

[145] Zayed A., Negm W., Kabbash A., Ezzat S.M. (2022). Marine-Derived Metabolites as Antimalarial Candidates Targeting Various Life Stages. J. Adv. Med. Pharm. Res..

[146] Singh H., Parida A., Debbarma K., Ray D.P., Banerjee P. (2020). Common Marine Organisms: A Novel Source of Medicinal Compounds. Int. J. Bioresour. Sci..

[147] Zhang M., Tian Z., Wang J., Tian X., Wang C., Cui J., Huo X., Feng L., Yu Z., Ma X. (2021). Visual Analysis and Inhibitor Screening of Leucine Aminopeptidase, a Key Virulence Factor for Pathogenic Bacteria-Associated Infection. ACS Sens..

[148] Fang C., Zhang J., Yang H., Peng L., Wang K., Wang Y., Zhao X., Liu H., Dou C., Shi L. (2019). Leucine Aminopeptidase 3 Promotes Migration and Invasion of Breast Cancer Cells through Upregulation of Fascin and Matrix Metalloproteinases-2/9 Expression. J. Cell. Biochem..

[149] Yang H., Dai G., Wang S., Zhao Y., Wang X., Zhao X., Zhang H., Wei L., Zhang L., Guo S. (2020). Inhibition of the Proliferation, Migration, and Invasion of Human Breast Cancer Cells by Leucine Aminopeptidase 3 Inhibitors Derived from Natural Marine Products. Anti-Cancer Drugs.

